# An Opto‐Actuated Hydrogel for Cell Mechanoactuation and Real‐Time Force Monitoring

**DOI:** 10.1002/advs.202511538

**Published:** 2026-01-04

**Authors:** Rinku Kumar, Marc A. Fernandez‐Yague, Adrien Bessaguet, Hosoowi Lee, Nicolas Giuseppone, Andrés J. García, Aránzazu del Campo

**Affiliations:** ^1^ INM – Leibniz Institute For New Materials Saarbrücken Germany; ^2^ SAMS Research Group, Université De Strasbourg, CNRS Institut Charles Sadron UPR 22 Strasbourg France; ^3^ Institut Universitaire de France (IUF) Paris France; ^4^ Petit Institute for Bioengineering and Bioscience Georgia Institute of Technology Atlanta Georgia USA; ^5^ Woodruff School of Mechanical Engineering Georgia Institute of Technology Atlanta Georgia USA; ^6^ Saarland University Chemistry Department Saarbrücken Germany

**Keywords:** cell forces, hydrogel, mechanoactuation, mechanotransduction, molecular motor, talin

## Abstract

Cellular force sensing and transduction are fundamental processes in development, homeostasis, and disease. To understand how cells detect and integrate mechanical forces, we need non‐invasive methods to apply forces at the molecular scale while monitoring cellular responses within physiological contexts. Here, we present a mechanoactuated hydrogel interface that can exert forces on integrin adhesion receptors and allows monitoring of traction force responses in real time. The actuation is achieved by light excitation of a rotary molecular motor presenting an adhesion peptide to bind integrins at the cell membrane and to a hydrogel surface via flexible polymer chains. Illumination results in chain twisting and an applied pulling force on the linked integrin receptors within subcellular illuminated areas. Fluorescent particles in the hydrogel allow parallel quantification of cellular forces by traction force microscopy. With this methodology, we monitored talin recruitment, actin organization, and traction force generation and their reversibility in response to applied forces by the rotary motor‐interface. We demonstrate reversible talin recruitment, enhanced F‐actin polymerization, and a reduction in cell traction force when force is applied to focal adhesions. This research expands the application of nano machine‐based actuation within soft hydrogels and showcases its capabilities.

## Graphical abstract

Researchers developed a mechanoactuated hydrogel interface where nanomachines exert forces on adhesion receptors and allow monitoring of traction force responses in real time. They demonstrate reversible talin recruitment, enhanced F‐actin polymerization, and a reduction in cell traction force when force is locally applied to focal adhesions of adherent cells.

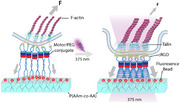

## 1 Main

Cells actively sense, integrate, and convert mechanical stimuli into biochemical signals [1]. Many processes in living organisms rely on mechanotransduction [2]. At the cellular level, focal adhesions (FAs) are pivotal nanoscale structures for mechanotransduction. FAs are molecular assemblies that physically connect the actin cytoskeleton to the extracellular matrix (ECM) and function as mechanosensors that regulate cellular responses to mechanical stimuli [3, 4, 5]. Talin, vinculin, integrins, and focal adhesion kinase (FAK) are key FAs components that undergo force‐induced conformational changes, modulating downstream signaling pathways [6]. Talin, for instance, unfolds under tension to expose vinculin‐binding sites. The actin cytoskeleton, through myosin‐II contractility, generates intracellular forces that are transmitted through FAs, with the magnitude of these forces being modulated by ECM mechanical properties [7, 8]. To quantify these forces, traction force microscopy (TFM) provides spatial and temporal maps of traction stress distribution [9]. An understanding of how force‐responsive mechanisms function at the molecular level and how they are coupled to force generation is important for elucidating cellular mechanotransduction pathways [7, 10, 11].

A major technical challenge for the investigation of mechanotransduction lies in achieving mechanoactuation with molecular specificity and spatiotemporal resolution, particularly when integrated with TFM. Several techniques have been developed to apply forces to individual FA molecules, such as magnetic tweezers, atomic force microscopy, and optical tweezers [12]. For instance, magnetic tweezers used to apply forces on purified talin revealed unfolding and exposure of hidden binding sites for vinculin [13]. A significant limitation of single‐molecule force spectroscopy techniques is that they necessitate specialized equipment capable of manipulating only one molecule at a time and often lack integration with cell traction force microscopy. An advantage of this technique is that the direction of the force application can be controlled. A different strategy for force application in cellular contexts involves the use of mechanically active materials for actuation. This approach leverages temperature‐responsive polymers and photothermal processes to facilitate actuation [14]. The mechanism of actuation is based on a polymer phase transition that results in either shrinking or swelling of a thin polymer layer. This property facilitates the exploration of dynamic processes with molecular precision. However, this approach has an inherent restriction in terms of spatial resolution [15] and the kinetics are limited by diffusion‐controlled swelling/deswelling of the polymer [16, 17]. Another strategy for force application involves photoswitchable hydrogels with photoactive yellow protein (PYP) based crosslinking, which enable up to 2 kPa cyclic changes in the hydrogel's Young Modulus at time scales of minutes [18]. This methodology has been successfully used to mechanically stimulate cells at a whole cell level. The potential extension of this principle to force application at subcellular spatial resolution has not been demonstrated.

**FIGURE 1:**
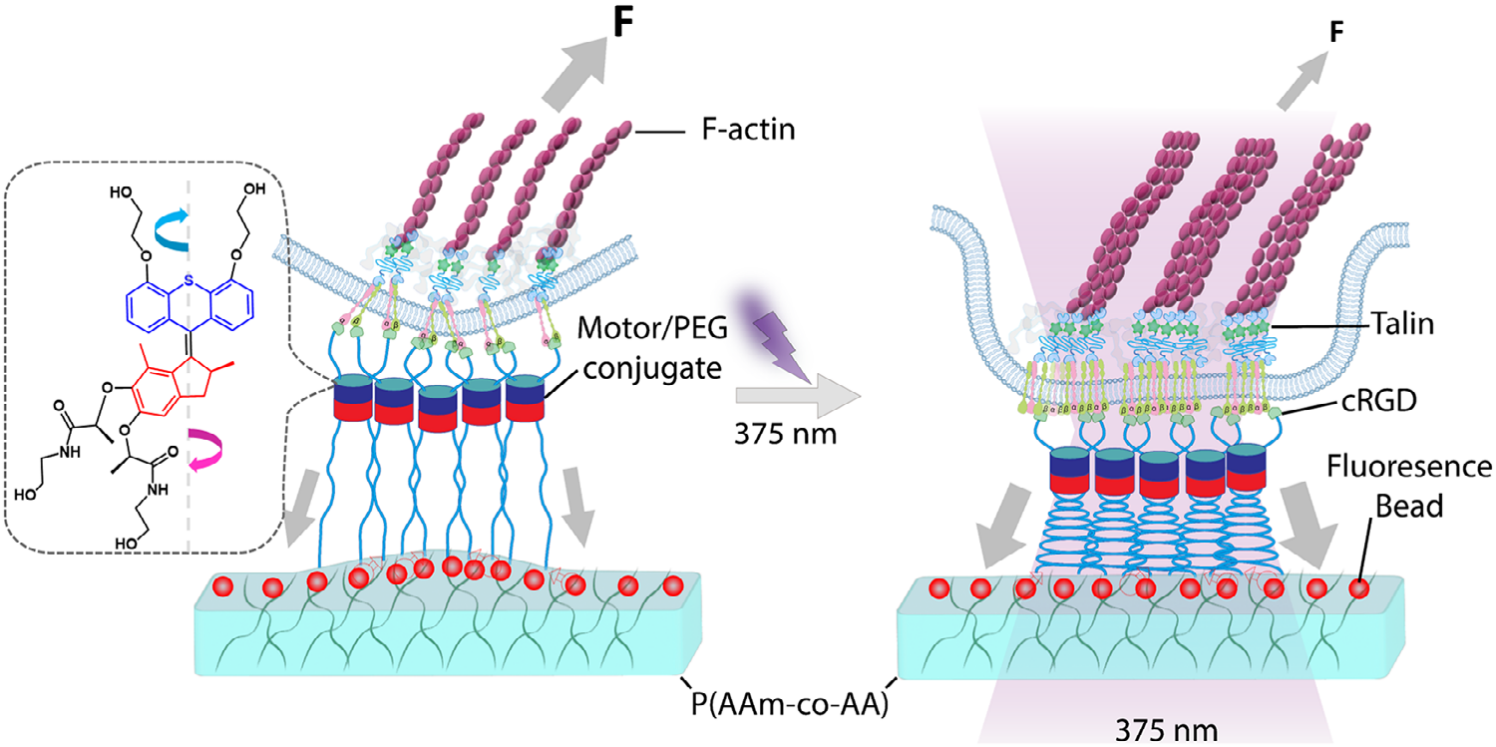
Scheme of mechanoactuated motor/PEG/RGD hydrogel platform adapted for traction force measurements. The motor/PEG/RGD conjugate is affixed to a polyacrylamide hydrogel on one end and to integrin on the opposite end. When subjected to localized illumination at 375 nm, the motor molecules undergo unidirectional twisting, leading to the entanglement of the elongated PEG chains and subsequently exerting a pulling force (opto‐actuate) on the FA assembly. The chemical structure of the motor is illustrated within the dotted box. Fluorescent beads embedded in the gel serve as markers for mapping the traction force applied by cells. In response to local opto‐actuation, the cell alters the internal dynamics of talin and F‐actin and modulates the traction force exerted externally.

In our prior research, we introduced a synthetic opto‐actuated interface [19]. By intercalating a light‐driven rotary molecular motor conjugated to a flexible PEG spacer between the adhesive receptors at the cell membrane and a non‐adhesive pegylated glass surface, we demonstrated local force application to FAs regulated with light exposure [19]. To advance the functionality of the optically actuated interface, we now conjugate the rotary motor to a soft hydrogel with cell‐relevant mechanical properties and enable monitoring and quantification of cellular traction forces by TFM in parallel to the force application. Figure 1 shows the working principle of the technique. The light‐triggered directional rotation of motor/PEG conjugates between the surface and the integrins at the cell membrane induces conformational twisting of the PEG chains, which converts the rotational motion into a contraction of the chain ensemble [20], and results in force application at the engineered biointerface. This entanglement results in an entropic force that possesses a potential tangential component, which emerges from the diminished configurational space following entanglement. This photoactuated interface allows spatiotemporal control of the force application, but in common with the other methods based on thermoresponsive polymers or molecular switches, it does not allow control of the direction of the applied force. It has been demonstrated that such a rotary motor can generate a force of approximately 20 pN [21, 22]. According to single‐molecule force spectroscopy studies, integrin–RGD bonds typically rupture under forces of 30–50 pN, while forces of ∼10–20 pN are sufficient to induce the conformational activation of integrins (switching from the bent to extended/open state) [13]. The threshold force of 20 pN seems, therefore, adequate to trigger integrin conformational changes in our experiments, potentially leading to integrin activation and subsequent downstream signaling molecules. In this report, we validate this methodology by monitoring talin recruitment, actin dynamics, and cellular traction forces alongside actuation.

## 2 Results

### 2.1 Development of a Mechanoactive Poly(acrylamide) Interface

Our elementary building block for actuation is a tetrapodal motor/PEG conjugate, which is coupled to a poly(acrylamide‐co‐acrylic acid) (P(AAm‐AA)) hydrogel on two terminals and to an integrin binding peptide (cyclicRGD, cRGD) for cell attachment on the other two end‐groups (Figure 1). The P(AAm‐AA) hydrogel contains embedded fluorescence particles for TFM. With this mechanoactive interface, we aim to apply forces to membrane integrins engaged in focal adhesions using light as an external stimulus while simultaneously imaging force‐responsive intracellular processes and quantifying traction forces in real time.

**FIGURE 2:**
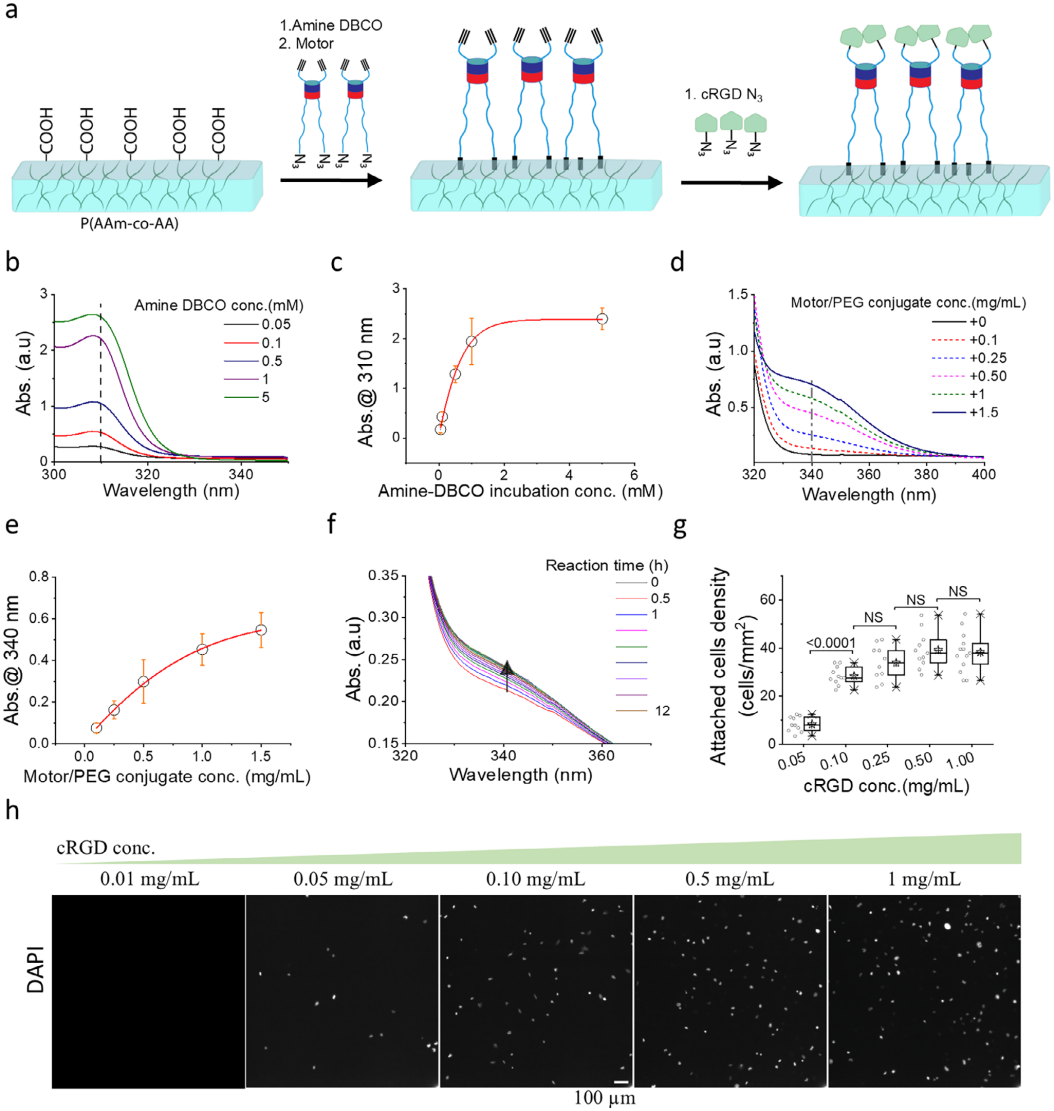
Preparation of the motor/PEG/RGD hydrogel for mechanoactuation and TFM. (a) Schematic of hydrogel functionalization with motor/PEG/RGD conjugation steps. (b,c) Quantification of amine‐DBCO coupling. (b) UV–vis spectrum of P(AAm‐co‐CC) hydrogel incubated with different concentrations of amine‐DBCO heterofunctional linker for 1 h. (c) The average absorbance value at 310 nm is represented as a function of Amine‐DBCO concentration. Data corresponds to three independent experiments and are fitted with an exponential function. Data represent mean ± s.d. from 3 independent experiments. (d–f) Quantification of the motor/PEG conjugate coupling step. (d) UV–vis spectrum of DBCO hydrogel reacted overnight with different concentrations of motor/PEG conjugate. (e). The corresponding average absorbance value at 340 nm as a function of motor/PEG conjugate concentration, incubated overnight. Data corresponds to mean ± s.d. from three independent experiments. Data were fitted with a rising exponential fit. (f) UV–vis spectrum of DBCO modified hydrogel reacted with motor/PEG conjugate (1 mg/mL) at increasing reaction times. (g) Box plot of cell density of talin‐YPet expressing mouse kidney fibroblast cells seeded on motor/PEG/RGD hydrogels incubated with RGD at concentrations ranging from 0.01 to 1 mg/mL for 1.5 h. Statistical significance was assessed by one‐way ANOVA followed by Tukey's multiple comparison test (*p*<0.0001; *p* = 3267; *p* = 3556; *p* = 0.9981). Data represent *n = 10, 11, 10, 11, and 13 fields of view* for each condition, respectively. Hydrogel was pre‐incubated with 1 mg/mL motor/PEG overnight and subsequently with RGD for 90 min prior to cell seeding. (h) Representative DAPI‐stained images of the cells on motor/PEG/RGD hydrogels incubated with RGD (0.01–1 mg/mL) are shown.

The hydrogel thin films (thickness ∼100 µm) are a copolymer of acrylamide (AAm), bis‐acrylamide crosslinker, and acrylic acid (AA). To attach the motor/PEG conjugate, the carboxylic groups of the P(AAm‐AA) hydrogel were activated with EDC/NHS chemistry, clicked to an amine‐dibenzocyclooctyne (DBCO) heterofunctional linker, and reacted with the azide‐terminated motor/PEG conjugate (Figure 2a; Figure S1a). Each reaction step was monitored by UV spectroscopy. The coupling of the amine‐DBCO linker was evidenced by the increase in the absorbance signal below 310 nm [23] when the AA of the hydrogel was reacted with various concentrations of amine‐DBCO (Figure 2b,c). The coupling of the motor/PEG conjugate was reflected in the increase in absorbance below 380 nm [22] (Figure 2d; Figure S1b) and in the decrease in absorbance at 310 nm as the triazole bond forms (Figure S1c). By adjusting the incubation concentration (Figure 2e) or the incubation time of the reactive molecule (Figure 2f; Figure S1c), hydrogels with different concentrations of motor/PEG conjugate were achieved. Unreacted DBCO groups were quenched by incubating with PEG‐azide solution (Figure S1d). In a last step, azide functionalized RGD adhesive ligand was coupled to the free alkyne ends of the motor/PEG conjugate by Cu catalyzed alkyne‐azide cycloaddition. As a control substrate, hydrogels were modified with a non‐rotary Episulfide motor/PEG conjugate (EpiSMot/PEG).

To validate the surface functionalization protocol and identify an appropriate ligand concentration for subsequent studies, talin1^−/−^ mouse embryonic kidney (MEK) fibroblasts stably re‐expressing talin1‐YPet at seeding density of 2.2 × 10^4^ cells/cm^2^ were incubated on the motor/PEG/RGD functionalized hydrogels at different RGD incubation concentrations for 2 h, and the attached cell density was quantified (Figure 2g,h). No cell attachment was observed on hydrogels that were not modified with RGD ligand (Figures S2 and S3), and an increase in the number of adherent cells was observed with increasing RGD incubation concentration up to 0.1 mg/mL (*p>0.0001*, Figure 2g). RGD concentrations higher than 0.1 mg/mL did not lead to a greater number of cells attached. These results confirm that the cells attach to the mechanoactive interface via the RGD peptide. Subsequent experiments were performed with hydrogels modified with 0.1 mg/mL RGD incubation concentration.

### 2.2 Light‐Actuated Motor/PEG/RGD Interface Enhances Local Recruitment of Talin and Reduces Cell Traction Forces

Our previous study using the motor/PEG/RGD conjugate coupled to ultrathin (<100 nm) PEG films on glass surfaces showed an increase in the total area of FAs within illuminated regions of interest (ROIs) in mouse embryonic fibroblasts stably expressing paxillin‐RFP [19]. We interpreted these results as FA reinforcement as a consequence of motor‐applied forces to the integrins engaged in FAs within the ROIs. In the present study with P(AAm‐co‐AA) hydrogels, we investigated whether forces exerted by the motor/PEG/RGD conjugate on integrins influence the recruitment of talin to FAs within the ROI using talin 1‐YPet expressing MEK fibroblasts. We also examined whether the exerted forces alter the organization of the F‐actin network and subsequently alter cell traction forces in MEKs by TFM. Talin 1 is a mechanosensitive protein that links integrins to the actin cytoskeleton [24, 25, 26]. It responds to mechanical signals originating from intracellular actomyosin contractility as well as external forces from the ECM and mediates force transmission between the ECM and the cytoskeleton [27, 28].

We seeded cells on the motor/PEG/RGD functionalized hydrogels at low density to have a large fraction of isolated cells which do not contact neighboring cells. For our experiments, we analyzed non‐polarized MEK cells, which exhibited circular morphology, with an average spread area of 1435 ± 360 µm^2^, perimeter of 137 ± 21 µm, circularity 0.95 ± 0.03, and an aspect ratio of 1.10 ± 0.07 across all experimental conditions (Figure S4). These cells represented ca. 50% of the cells within the culture plate (data not shown). This selection was motivated by the requirement that cells remain stably positioned within the UV illumination ROI during the 35‐min optoactuation experiment. Polarized fibroblasts with an elongated morphology are typically migratory and move out of the ROI within this timeframe. We used a 375 nm Opti‐MicroScan laser to photoactivate the rotation of the motor within ROIs. We also used the FITC channel to monitor talin 1‐YPet localization and the TRITC channel for imaging the displacement of red fluorescent particles by TFM.

For localized opto‐actuation, two circular ROIs with a diameter of 7 µm (ROI area 38 µm^2^) and encompassing 1–5 FAs were illuminated. The selected ROIs were positioned at diagonal locations at the cell periphery (Figure S5a, Movie S1). Laser power density and pulse duration are critical parameters for the opto‐actuation, and these parameters were optimized prior to the experiments [19]. The turnover time of individual FAs, reported on the scale of tenths of minutes [29, 30, 31], constitutes a relevant boundary condition for our experiments. Consequently, we selected an experimental window of 30 min. A minimum number of 3 images per minute were necessary to unequivocally track the evolution of individual FAs during the experiment. With our microscopy setup, 6 s were required to capture fluorescence images from two channels (talin and beads). With these boundary conditions, we set a UV illumination time of 6 s for each ROI. Both ROIs were illuminated consecutively, and fluorescence images of each channel (talin and beads) were captured in between. With this configuration, we adjusted the laser power density to minimize cellular damage, which we evaluated by monitoring the retraction of the cell edge at the illumination site and the change on overall spread area. We found exposure greater than or equal to 74 mW.cm^−2^ led to retraction of the cell edge at illuminated ROI and reductions in total spread area (Figure S5a–c). Higher laser power density led to a sudden breakdown of FAs and a significant decrease in the cell spread area. Exposure to 49 mW.cm^−2^ laser power density neither caused retraction of the cell edge, nor a change in the spread area of the cells within the 30 min experimental time. Live/dead assays confirmed the absence of phototoxicity at these exposure conditions (experiments described below). We subsequently tested potential temperature changes at ROIs during exposure using Rhodamine B as temperature sensitive dye (Figure S6). Illumination at power densities <98 mW.cm^−2^ did not lead to significant temperature variations at the illuminated site. All subsequent experiments were performed at 49 mW.cm^−2^ laser power density.

Initially, we aimed to replicate our previous findings that force application increases the area of FAs. Under optimized illumination conditions, two ROIs, designated as ROI1 and ROI2 (Figure 3a, red circles), were sequentially exposed to UV illumination for 30 min. A third ROI, designated ROI3 (Figure 3a, green circle), was randomly selected at the cell periphery, far away from ROI1 and ROI2. ROI3 was not illuminated and served as an internal control to assess the effects of optoactuation on not exposed cellular regions during the experiment. We first tracked the area of the individual FAs within the ROIs of talin1‐YPet‐expressing MEK fibroblasts (Figure S7a,b). Segmentation by image analysis indicated an increase in the area of individual FAs within illuminated ROIs (ROI1, *p = 0.0455*; ROI2, *p = 0.0455*) whereas no significant changes were observed in the non‐irradiated ROI3 (*p = 0.9552*, Figure 3a,b; Movie S1). Small nascent FAs that appeared and disappeared during tracking were excluded from this analysis (Movie 1). These results indicate that the motor‐applied forces induce a local increase in the area of individual FAs, corroborating our earlier findings with paxillin‐RFP‐expressing fibroblasts [19].

FIGURE 3Talin and cell force response to mechanoactuated hydrogel. (a) Representative invert image of a talin‐YPet expressing cell attached to the motor/PEG/RGD hydrogel interface. FAs are labeled with talin‐YPet. Two diagonally arranged ROIs marked in red were illuminated, while the ROI in green was not illuminated. The magnified, color‐coded FAs area images of ROI1, 2, and 3 show the evolution of the size of FAs in each region during the 30‐min experiment. (b) Relative increase in average focal adhesion area of ROI1, 2, and 3 measured through tracking of FAs. Data were overlayed with fitted four‐point smoothening. (c) Fluorescence microscopy images of talin (T) and corresponding traction force (F) map of a cell on the motor/PEG/RGD interface at different time points. The bottom section displays cropped data for Talin and traction force from ROI 1 (T1, F1) and 2 (T2, F2). (d) A line scatter plot illustrating the average T intensity alongside the corresponding average F for each ROI 1–3 throughout the experiment. The error bars indicate the standard deviation, and the shaded area highlights the time duration of the active 375 nm laser. Shaded regions in the plot show UV exposure duration. (e,f) Identical experiments presented in (c,d) but using the control EpiSMotor/hydrogel interface. (g) A box scatter plot illustrating the relative values of Talin intensity at the 10‐min interval is presented for both the EpiSMot and Motor/PEG/RGD interfaces, as well as for the Motor/PEG/RGD interfaces subjected to a 1‐h pre‐treatment with 5 µm Y27632 (Y27) after UV‐mediated motor activation. Data represents FAs tracks gathered from n = 28,11,16,28,11,16,28,11, and 16 cells pooled from over 3 independent experiments. Statistical analysis was performed by one‐way ANOVA with Bonferroni multiple comparison test: ROI1(EpiSMot vs. Mot, *p*<0.0001, EpiSMot vs. Mot+Y27, *p*<0.0001; Mot vs. Mot+Y27, *p* = 0.9748), ROI2(EpiSMot vs. Mot, *p*<0.0001; EpiSMot vs. Mot+Y27, *p*<0.0001; Mot vs. Mot+Y27, *p* = 0.9999), ROI3(EpiSMot vs. Mot *p*<0.0001; EpiSMot vs. Mot+Y27, *p*<0.0001; Mot vs. Mot+Y27, *p* = 0.9748). (h) The talin intensity of motor/PEG/RGD conjugate was modeled using an exponential rise described by the equation y(t) = A(1‐e^(‐bt)) where y(t) denotes the talin fluorescence intensity, A indicates the maximum amplitude, b represents the rate constant, and t is the time in minutes. The rate constant and talin intensity amplitude for all data presented in panel d corresponds to regions of interest ROI 1, 2, and 3. Statistical analysis was performed using one‐way ANOVA with Tukey's multiple comparison test: Rate constant (ROI1 vs. ROI3, *p*<0.0001; ROI2 vs. ROI3, *p*<0.0001; ROI1 vs. ROI2, *p* = 0.8763). Amplitude (ROI1 vs. ROI3, *p*<0.0001; ROI2 vs. ROI3, *p*<0.0001; ROI1 vs. ROI2, *p* = 0.9763). (i) A corresponding box scatter plot depicting the relative traction force values 10 min after UV‐mediated motor activation is provided for the EpiSMot, Motor/PEG/RGD interfaces, and the Motor/PEG/RGD interfaces pretreated with Y27632 (Y27) for 1 h. Data represents FAs tracks collected from n = 28,11,16,28,11,16,16,28,11, and 16 cells across > three independent exp. Statistical analysis was performed using one‐way ANOVA with Bonferroni multiple comparison test: ROI1(EpiSMot vs. Mot, *p*<0.0001;EpiSMot vs. Mot+Y27, *p* = 0.9748; Mot vs. Mot+Y27, *p*<0.0001), ROI2(EpiSMot vs. Mot, *p*<0.0001;EpiSMot vs. Mot+Y27, *p* = 0.6417; Mot vs. Mot+Y27, *p*<0.0001), ROI3(EpiSMot vs. Mot, *p*<0.0001;EpiSMot vs. Mot+Y27, *p* = 0.001; Mot vs. Mot+Y27, *p* = 0.001).

**Figure:**
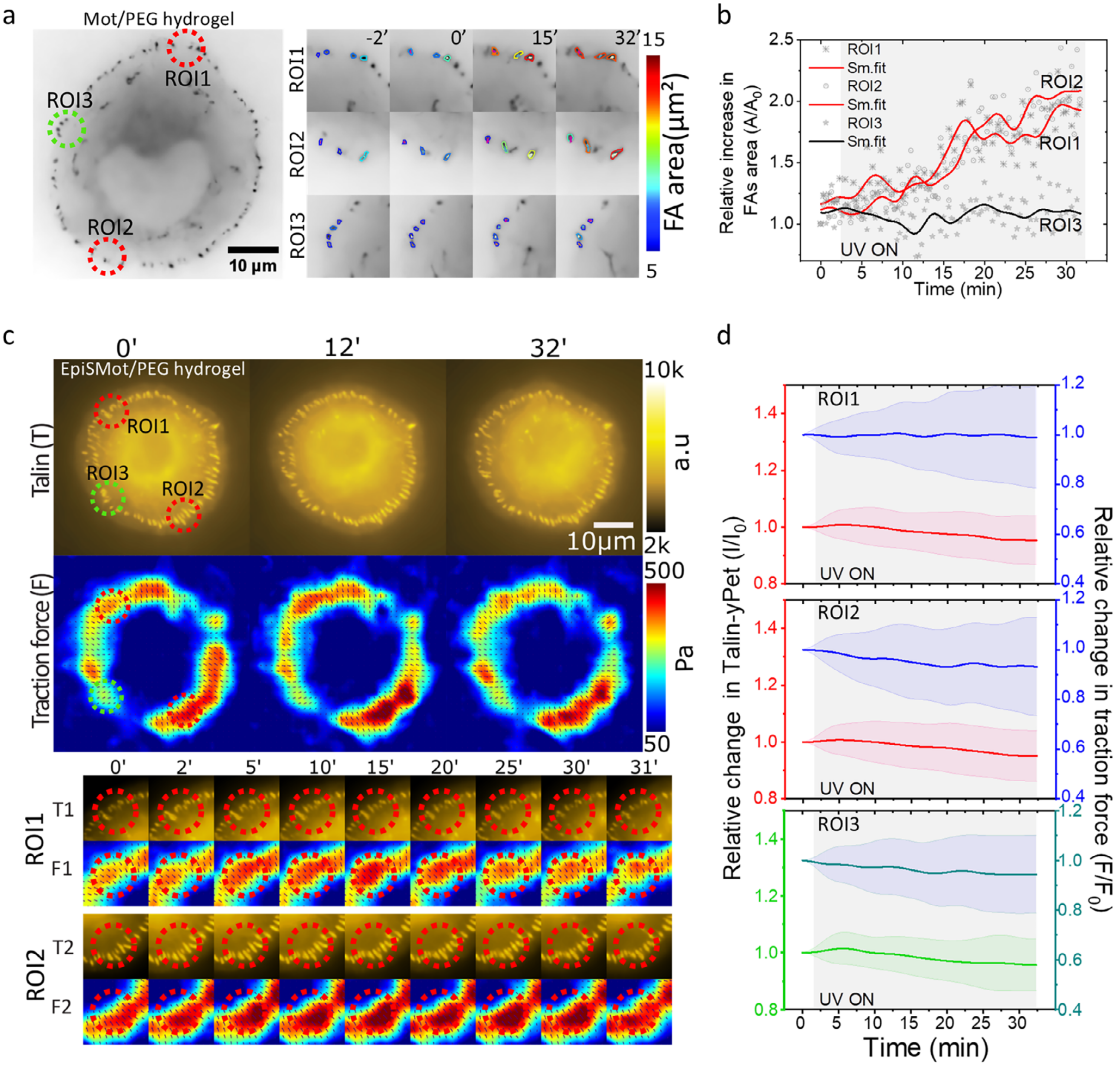

**Figure:**
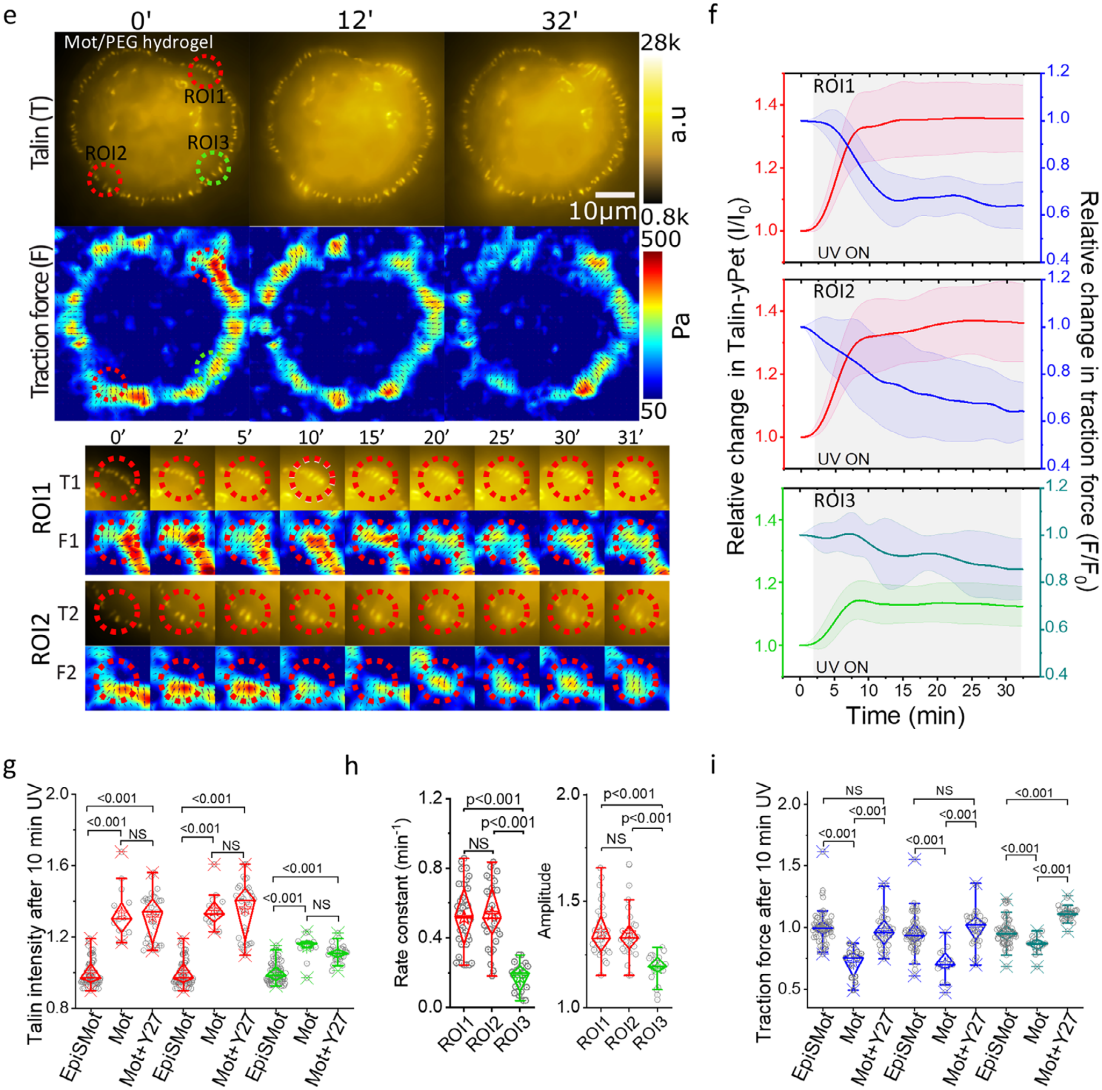

Next, we investigated the behavior of talin under force application to FAs, focusing on the temporal dynamics of the average talin signal (average intensity across all segmented pixels) at individual focal FAs within the ROIs. Initially, we assessed the impact of illumination on FAs of cells situated on a non‐rotatory EpiSMot/PEG/RGD control surface. Over a 30‐min period, we found no significant alterations in talin intensity in either the illuminated or non‐illuminated ROIs (Figure 3c,d; Movie S2). We conducted similar measurements on cells adhering to a rotatory motor/PEG/RGD surface, where we observed a rapid exponential increase in talin intensity within the first 5 min of illumination, followed by a plateau at the 10‐min mark in both illuminated and non‐illuminated ROIs (Figure 3e,f; Movie S3). Therefore, for the purpose of statistical analysis, we utilized the change observed after 10 min as a reference point to evaluate the impact of opto‐actuation. For cells adhering to the rotatory motor/PEG/RGD surface, we observed significant and equivalent increases in talin intensity at FAs for both illuminated ROI 1 and 2 (Figure 3g). A smaller but significant increase in talin intensity was observed for FAs in the non‐illuminated ROI3. We attribute this increase in talin signal to the reaction forces needed to maintain the cell in mechanical equilibrium following mechanoactuation. In contrast, all ROIs in the control EpiSMot/PEG/RGD surface exhibited no changes in talin intensity (Figure 3g).

We also monitored talin recruitment at longer optoactuation times up to 120 min. Talin intensity remained sustained at the illuminated ROIs (Figure S8) for approximately 70 min. A small decline in talin intensity was observed between 70‐ and 120‐min illumination (Figure S8b). We attribute this decay to photodamage during imaging. To further assess photodamage, we performed a live/dead assay using the fluorescent dye SYTOX Deep Red, which accumulates at the cell nucleus upon membrane damage (Figure S8c,d). We did not observe a significant accumulation of SYTOX Deep red in illuminated cells up to 120 min compared to dead cell controls (Figure S8e,f), indicating that overall cell viability is preserved under our experimental conditions.

To analyze the changes in talin fluorescence intensity, we employed an exponential rise model represented by the equation *y*(*t*)  =  *A*(1 − *e* ^−*bt*^) where *y(t)* denotes talin fluorescence intensity, *A* indicates the maximum amplitude, *b* represents the rate constant, and *t* is the time in minutes. Upon fitting the model, we noted a 35% increase in talin intensity amplitude (ROI1, *A*  =  1.36 ± 0.12; ROI2, *A*  =  1.35 ± 0.13) during the first 10 min of illumination, followed by a plateau over the subsequent 20 min (Figure 3e,f; Movie S3). In contrast, the non‐illuminated area ROI3 exhibited a similar pattern, albeit with only a 19% increase, which also stabilized (ROI3, *A*  =  1.19 ± 0.07) (Figure 3h). Furthermore, the rate of increase, denoted by *b*, was notably faster (p *< 0.001* for both) in the illuminated ROI1 (*b*  =  0.52 ± 0.24) and ROI2 (*b*  =  0.54 ± 0.18) compared to the non‐illuminated ROI3, *b*  =  0.17  ±  0.04) (Figure 3h). This result indicates that the application of force on integrins increases the recruitment rate of talin to FAs.

Talin is essential for linking actin filaments within the cytoskeleton to integrins located at the cell membrane, thus enabling the transmission of forces in both directions to the extracellular matrix. We next explored the relationship between talin recruitment to FAs and the traction force during the application of forces to FAs. We therefore established and validated TFM protocols in our hydrogels. Initially, we conducted experiments to rule out the possibility of substrate deformation caused by a 375 nm laser on the motor/PEG/RGD hydrogel. To achieve this, we monitored the movement of beads during the illumination of the motor/PEG/RGD conjugate hydrogel in the absence of cells throughout the experiments, while varying the laser power density. We found no significant deformation within the laser power density range of 49–392 mW.cm^−2^ (Figure S9). Photobleaching of bead fluorescence occurred at laser power density exceeding 392 mW.cm^−2^, which is well above the conditions used in our experiments. We also performed experiments to rule out a possible alteration of the mechanical properties of the motor/PEG hydrogel during illumination. Rheological measurements of pristine, amin‐DBCO, and motor/PEG functionalized hydrogels were performed (Figure S10). No significant differences were observed in the storage (G′) and loss (G″) moduli during 10 min UV illumination among the hydrogels, confirming that UV exposure does not affect their macroscopic mechanical properties.

Next, we confirmed the TF measurement method by monitoring and analyzing the migration patterns of polarized and non polarized cells on the motor/PEG/RGD conjugate and compared the generated traction maps with the corresponding migratory/non migratory behaviour and shapes of the cells (Figure S11). We also assessed the reliability of the traction force reconstruction algorithm by normalizing the bead displacement data against the traction force (Figure S12).

We then examined the correlation between talin recruitment and traction force during the application of forces to FAs within the same ROI. To facilitate this analysis, we utilized segmented ROIs derived from the talin‐YPet channel and superimposed them onto the traction stress map. We also explored the temporal evolution of the average talin signal in relation to the average traction force at individual FAs within the illuminated ROIs. Unexpectedly, we noted a gradual decrease in traction force up to 35% in both illuminated and non‐illuminated ROIs (Figure 3f,i) as the intensity in the talin signal increased. We examined the contributions of actomyosin contractility to these responses. Rho‐associated protein kinase (ROCK) serves as a primary regulator of the actomyosin network [32, 33]. Therefore, we repeated the experiment after inhibiting ROCK activity using 5 µm Y27632 treatment for 1 h. Cells treated with Y27632 experienced a significant reduction in basal traction force (Figure S13a,b), accompanied by a significant increase in cell spread area and perimeter, (Figure S4), while circularity and aspect ratio remained unaltered. When motor‐induced forces were applied to the focal adhesions of ROCK‐inhibited cells, talin recruitment persisted without changes in traction force (Figure 3i; Movie S4), indicating that changes in traction force in response to force application require actomyosin contractility. These data support a model in which the application of force on integrin‐talin complexes activates downstream ROCK signaling, which subsequently reduces the contractility of the actomyosin network, resulting in a decrease in traction force.

### 2.3 Mechanoactuation of Integrins Enhances F‐Actin Polymerization

Traction force arises from the contractile force that cells exert on the ECM by their intracellular structural framework, including F‐actin, myosin motor proteins, talin, and integrins connected to the ECM. We investigated whether the force applied to the ROI led to changes in the F‐actin network. We labeled MEK fibroblasts with the F‐actin binding dye FastActSPY650 and examined temporal changes in the average talin signal in relation to F‐actin at FAs within the illuminated ROIs.

We first performed experiments to apply force on the non‐rotatory EpiSMot/PEG/RGD interface. The status of F‐actin was assessed by overlaying the talin region of interest with the F‐actin channel. Our findings indicated no notable changes in talin recruitment or F‐actin polymerization in either the illuminated (ROI1 and ROI2) or non‐illuminated (ROI3) FA regions (Figure 4a,b; Movie S5).

FIGURE 4Talin and F‐actin responses to mechano‐actuated interface. (a) The image presented below depicts the Talin with corresponding F‐actin labeled with FastActSPY650 of the same cell that is attached to the EpiSMotor/PEG/RGD hydrogel interface, with observations documented over the experiment duration. Two red ROIs highlight the areas that were subjected to illumination, while the third green ROI denotes a non‐illuminated area, utilized for assessing Talin and the related F‐actin intensity. The upper section features cropped data for Talin (T) and F‐actin (A) from regions 1 (T1, A1), 2 (T2, A2) as illustrated in the image below. (b) The line scatter plot displaying the average intensity of talin in relation to the corresponding F‐actin for each illuminated ROIs over the course of the experiment. Error bars represent the standard deviation, and the shaded region indicates the duration of active 375 nm laser exposure. (c,d) The information provided in is the same as a‐b, with the only difference being the cells cultivated on the Mot/PEG/RGD hydrogel interface. (e) A box scatter plot presents the relative values of talin intensity in ROIs 1, 2, and 3, measured 10 min after the activation of the motor via UV light. Data shows FAs tracks collected from n = 11,12,11,12,11 and 12 cells from more than three independent exp. Statistical analysis was performed using the Mann–Whitney U‐test: ROI1(EpiSMot vs. Mot, *p*<0.0001), ROI2(EpiSMot vs. Mot, *p*<0.0001), ROI3(EpiSMot vs. Mot, *p*<0.0001). (f) The talin intensity of Mot/PEG/RGD conjugate was modeled using an exponential rise described by the equation y(t) = A(1‐e^(‐bt)) where y(t) denotes the talin fluorescence intensity, A indicates the maximum amplitude, b represents the rate constant, and t is the time in minutes. The rate constant and talin intensity amplitude for all data presented in panel d corresponds to regions of interest ROI 1, 2, and 3. Statistical analysis was performed using one‐way ANOVA with Tukey's multiple comparison test: Rate constant (ROI1 vs. ROI3, *p*<0.0001; ROI2 vs. ROI3, *p*<0.0001; ROI1 vs. ROI2, *p* = 0.8763). Amplitude (ROI1 vs. ROI3, *p*<0.0001; ROI2 vs. ROI3, *p*<0.0001; ROI1 vs. ROI2, *p* = 0.9763). (g) A box scatter plot presents the relative values of traction force in ROIs 1, 2, and 3, measured 10 min after the activation of the motor via UV light. Statistical analysis was performed using the Mann–Whitney U‐test: ROI1(EpiSMot vs. Mot, *p*<0.0001), ROI2(EpiSMot vs. Mot, *p*<0.0044), ROI3(EpiSMot vs. Mot, *p*<0.0001).

**Figure:**
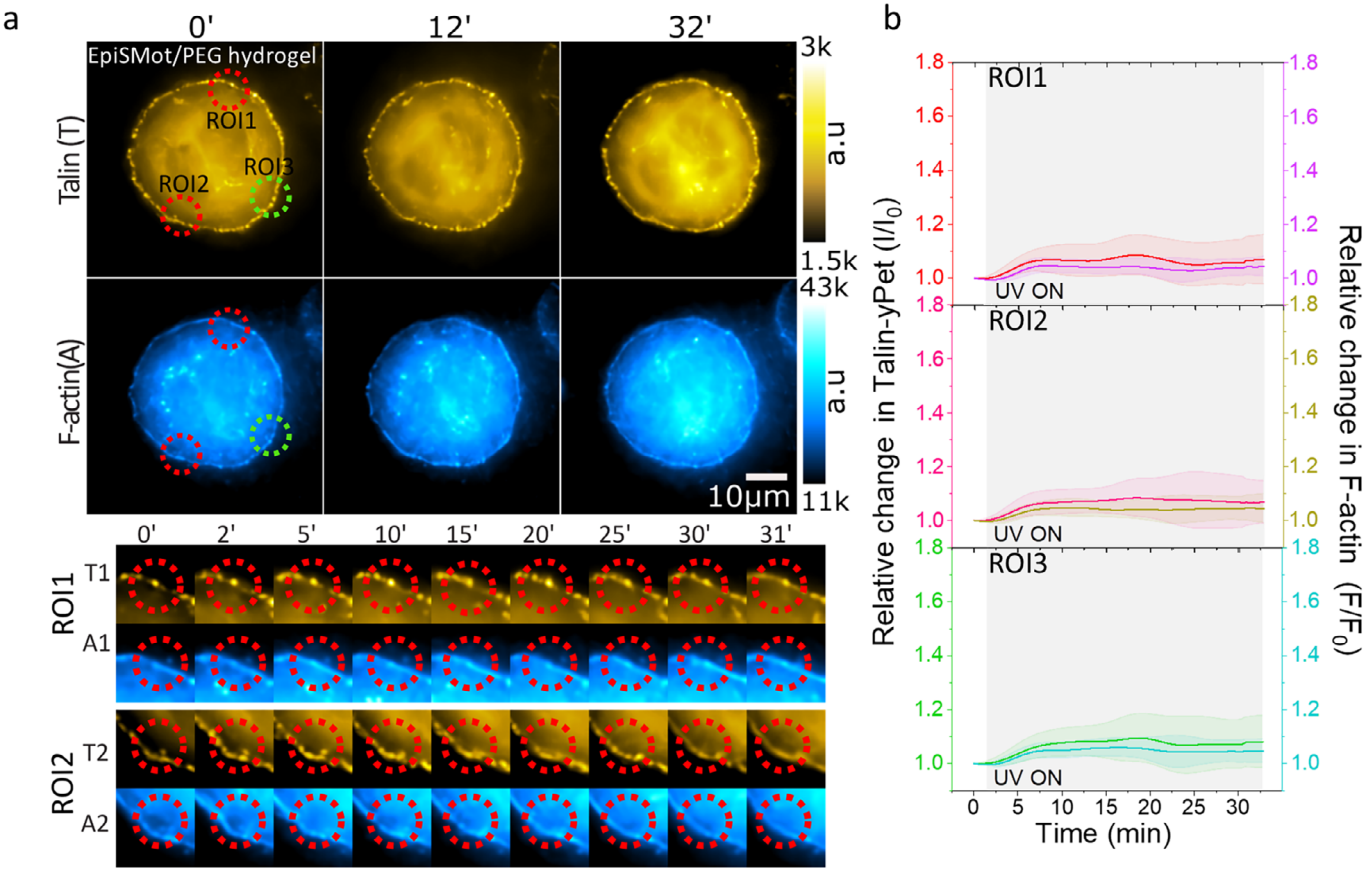

**Figure:**
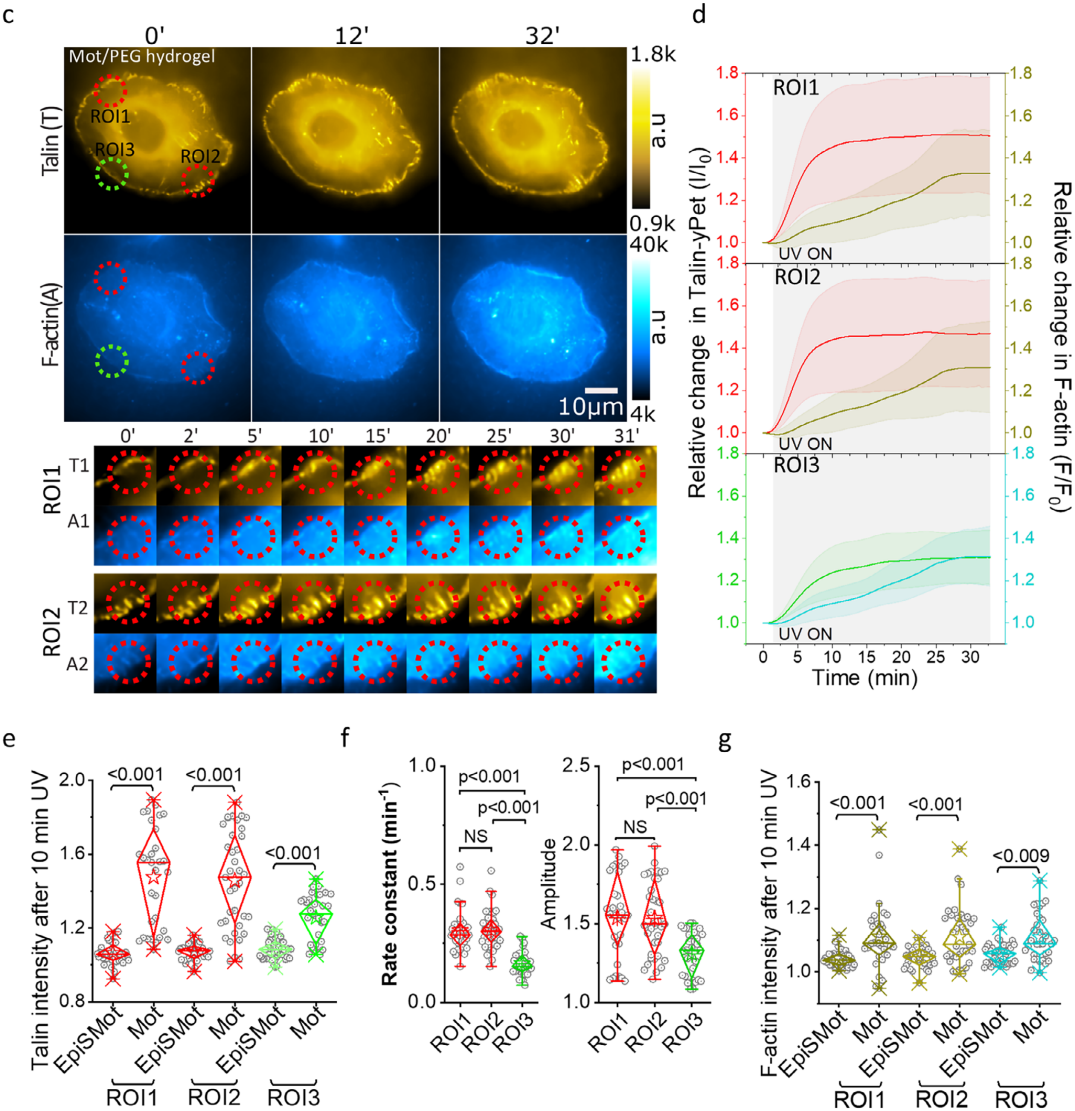

For the motor/PEG/RGD interface, we observed a rapid exponential increase in talin intensity within 5 min after mechanoactuation of FAs in the designated illuminated ROI1 and ROI2, subsequently followed by a phase of stabilization (Figure 4c,d; Movie S6). The corresponding F‐actin intensity shows a linear increase over the 30‐min imaging window.

We conducted a similar analysis of talin fluorescence intensities utilizing an exponential rise model, as previously described. Upon fitting the data to the model, we observed a 53% increase in the amplitude of talin intensity (ROI1, *A*  =  1.54 ± 0.26 and ROI2, *A*  =  1.53 ± 0.23) during the first 10 min of illumination, followed by a plateau over the subsequent 20 min. The non‐illuminated area ROI3 exhibited a similar pattern, albeit with only a 30% increase, which also stabilized (ROI3, *A*  =  1.30 ± 0.14) (Figure 3h). Furthermore, the rate of increase (*b*), was notably faster (*p < 0.001* for both) in the illuminated ROI1 (*b*  =  0.30 ± 0.09) and ROI2 (*b*  =  0.30 ± 0.08) than in the non‐illuminated ROI3, *b*  =  0.16  ±  0.05) (Figure 4e,f).

We evaluated the effects of opto‐active stimulation on changes in F‐actin intensity in illuminated areas 1 and 2 after 10 min of illumination. We found a significant increase in F‐actin intensity at the rotating motor interface compared to the control (non‐rotating motor) interface (Figure 4g). In contrast to talin, the F‐actin response was similar between illuminated and non‐illuminated ROIs (ROI1,1.13±0.08; ROI2,1.11±0.09; ROI3,1.11±0.06). The increase in F‐actin within non‐illuminated areas suggests that mechanical loading of FAs triggers downstream signaling pathways, including those associated with Rho GTPases (such as RhoA), which play a role in regulating the polymerization of the actin cytoskeleton, thereby enhancing the production of F‐actin filaments. However, an in‐depth examination of Rho GTPase activation falls outside the parameters of this study. This observation indicates that the actuation of FAs promotes localized recruitment of talin and enhances the overall polymerization of F‐actin within the cell.

### 2.4 Cell Response to Force Relaxation

**FIGURE 5:**
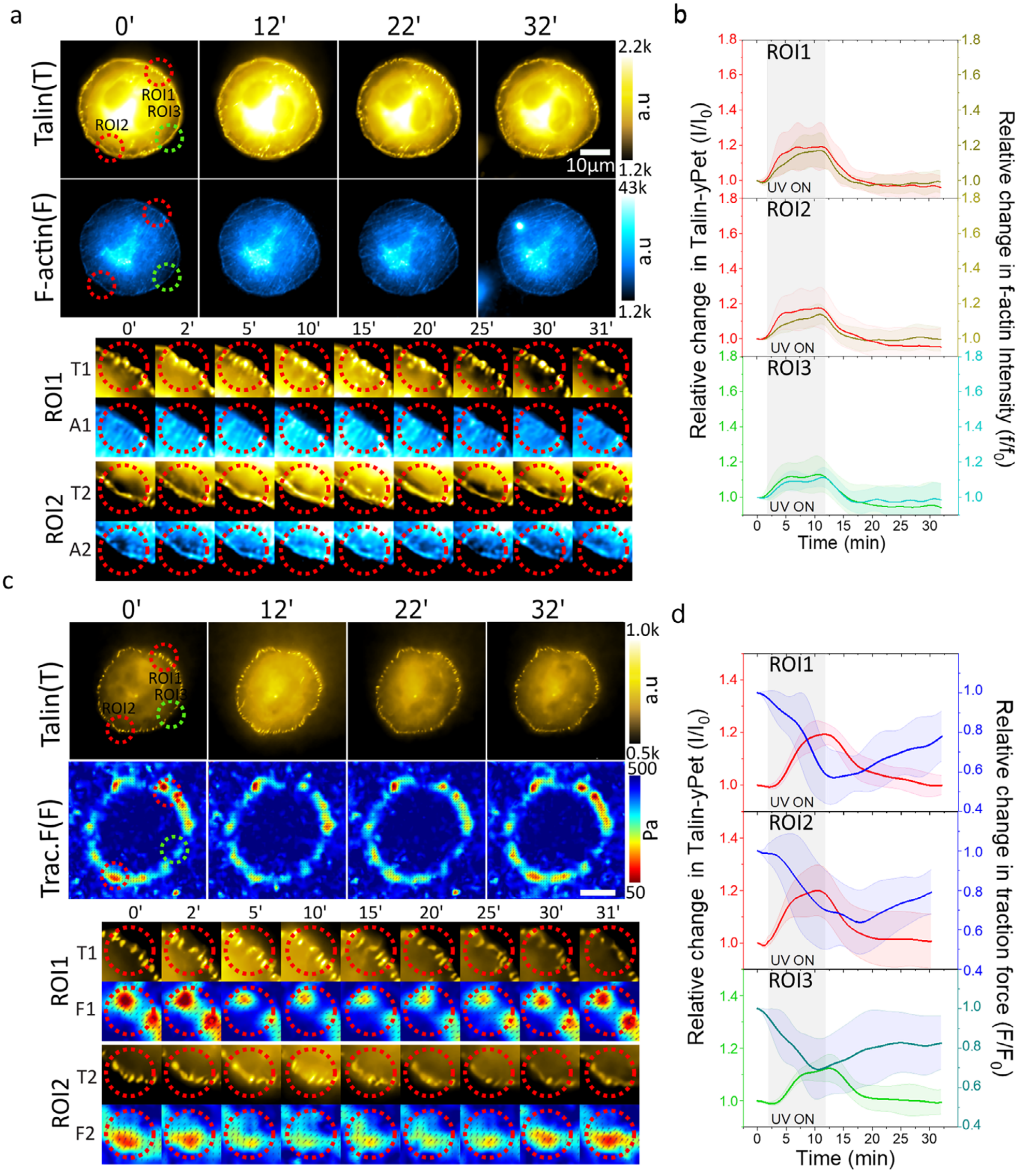
Cell responses to force relaxation. (a) The image displayed illustrates the Talin, along with the corresponding F‐actin labeled with FastActSPY650, from the same cell that is in contact with the Motor/PEG/RGD hydrogel interface. Two red dotted ROIs are marked to indicate the areas that received illumination, while a third green ROI represents a non‐illuminated area, which was used to evaluate the intensity of Talin and the associated F‐actin. The time noted at the top reflects the duration of the experimental observations. The lower section contains cropped data for Talin (T) and F‐actin (A) from regions 1 (T1, A1) and 2 (T2, A2), as depicted in the image below. (b) The line scatter plot illustrates the average intensity of talin in relation to the corresponding F‐actin for each illuminated region of interest (ROI) throughout the experiment. The error bars denote the standard deviation, while the shaded area signifies the period of active exposure to the 375 nm laser. Data represent mean ± s.d. (n = 8 cells each from three independent exp). (c) The images depict the talin focal adhesion in conjunction with the corresponding traction map of a cell situated on the motor/PEG/RGD interface, with observations documented over a specified time period. Two dotted red ROIs highlight the illuminated areas, while the third green ROI signifies a non‐illuminated region utilized for assessing Talin and the related local traction force. The lower section presents cropped data for Talin (T) and traction force (F) from ROI 1 (T1, F1) and ROI 2 (T2, F2). (d) Line scatter plot illustrates average talin intensity and corresponding average traction force for ROI (1‐3) across the experiment. Error bars represent mean ± s.d. (n = 4 cells from three independent exp). The shaded regions indicate the duration of 375 nm laser activation (UV exposure).

The rotation of the motor molecule, which leads to the coiling of the PEG chains in the motor/PEG conjugate, occurs exclusively during light exposure. Therefore, we anticipate that the applied force will diminish in the absence of illumination as the chains revert to their relaxed state. This relaxation has been demonstrated in mechanoactuated gels [20, 22], and its timescale depends on the number of turns in the motor/PEG/RGD conjugate. We monitored the cellular response after turning off illumination to assess the potential reversibility of the mechano‐actuated cellular response. In our previous experiments, we had observed that talin recruitment reached saturation after 10 min of force application on FAs. Thus, we subjected FAs in ROI1 and ROI2 to 10 min of exposure before discontinuing illumination. We then tracked the recruitment of talin and polymerization of F‐actin at FAs in the illuminated and non‐illuminated ROIs over a 30‐min period. We observed a rise in talin recruitment and polymerized F‐actin during the first 10 min of illumination in UV‐exposed FAs (ROI1 and ROI2), followed by an exponential decay to baseline levels after discontinuation of illumination (Figure 5a,b; Movie S7). The non‐illuminated ROI (ROI3) exhibited a similar trend, although the peak of talin recruitment was lower than that observed in the illuminated ROI (ROI3). This outcome indicates a positive and reversible correlation between the responses of talin and F‐actin to the force exerted on the FAs.

Additionally, we examined the talin signal and the traction force during the illumination and subsequent non‐illumination period. An initial exponential increase in the talin signal coincided with a decrease in the traction force during the first 10 min in the illuminated FAs (Figure 5c,d). After discontinuation of illumination, the talin signal decayed to baseline levels over the next 20 min, while the traction force increased over time. A similar but less pronounced trend was noted in the traction force at the non‐illuminated ROI (ROI3). In a 6‐steps photoactuation cycle with on/off intervals of 10 min, Talin recruitment was found to increase and decrease following the illumination cycles (Figure S14a). A weak oscillation of talin intensity was also observed in non illuminated ROI. We noticed a decay in the talin signal within the illuminated ROIs after 80 min and in the non‐illuminated areas. This suggests a potential photobleaching of the sample during the imaging cycle. Deep Red staining of the cells did not show significant membrane damage under these conditions (Figure S14a,c), indicating that cell viability was maintained during the long‐term cyclic mechano‐actuation.

In summary, these results suggest that the application of force to FAs through our optomechanical interface stimulates talin recruitment and F‐actin polymerization, thereby influencing the overall mechanical state of the cells in a reversible manner.

## 3 Discussion

In our prior work, we reported a synthetic biointerface designed for the opto‐actuation of individual integrins [19]. In the present study, we expand this approach to a soft hydrogel substrate with the capability to monitor traction forces in parallel to the optoactuation. We developed a coupling protocol that allows spectroscopic monitoring of the coupling steps and selective attachment of cells through adhesive ligands bound to the motor. The experiments in our study were performed in 2 h to avoid the possibility of fibronectin deposition and preserve the molecular specificity of the mechanoactuation.

The mechanoactuated hydrogel interface allowed quantitative monitoring of talin recruitment. Talin accumulates at FAs in a manner that is responsive to the stiffness of the substrate [26, 34]. Using the mechanoactuated hydrogel, we monitored the real‐time recruitment of talin to FAs within minutes after localized opto‐actuation at ROIs. The increase in talin levels reaches saturation within 10 min, aligning with the reported turnover time of many FA proteins, and indicating a potential interconnection among adhesion proteins at FAs [29, 30, 31]. The recruitment kinetics of talin in areas subjected to applied force are more rapid than in regions without such force. Furthermore, cells with inhibited ROCK activity exhibit comparable levels of talin recruitment following integrin opto‐actuation, indicating that externally applied force alone is sufficient to trigger talin recruitment. Integrins connect the actin cytoskeleton to the ECM through talin and play a crucial role in the bidirectional transmission of mechanical forces. Our study revealed that local integrin activation could partially propagate to other regions of the cell (comparing illuminated and non‐illuminated ROIs), resulting in a reversible decrease in overall cellular traction force. Furthermore, the application of a ROCK inhibitor eliminates the decrease in traction force during FA force application. This inverse relationship between talin recruitment and the reduction in traction force stands in contrast to prior studies that have reported a positive correlation among stiffness, traction force, and talin intensity [35, 36, 37]. Recent work also describes an enhancement in cell traction forces in response to cyclic dynamic mechanical stiffening of the substrate in the 1–5 min time scale [18]. A potential explanation for our observation is that local loading of FAs during illumination leads to rupture of integrin‐RGD bonds, resulting in decreases in local traction force. This explanation is consistent with the observed increases in traction force as the illumination was discontinued, as new bonds would form without load‐induced rupture. This explanation suggests a biophysical limit to integrin‐mediated mechanotransduction where there is an optimal range of force that promotes adhesion, and exceeding this range undermines the stability of adhesive contacts. With the present results, the specific mechanism behind the reduced traction force cannot be unraveled. Future high‐resolution imaging experiments during opto‐actuation may reveal if illumination promotes local heterogeneous assembly of F‐actin through ROCK activation, leading to a reduced traction force.

In the present study, we cannot exclude small changes in the nanoscale spacing of the RGD adhesive ligand during actuation, which could impact integrin clustering, FA formation, and cell traction forces at spacings within the 70–150 nm range [38]. A motor‐driven clustering of RGD‐integrin complexes could lead to reduced inter‐ligand spacing and contribute to mechanotransduction by promoting cytosolic F‐actin polymerization [39]. Further work is needed to confirm the underlying mechanism(s).

Nevertheless, our findings align with previous observations made using a cell stretching apparatus to apply forces on integrins. The stretching of focal adhesions leads to an increase in both the area and tension within the vinculin protein [40, 41, 42], accelerated F‐actin polymerization [40, 43], and simultaneously softened F‐actin [34, 44, 45], leading to a reduction in cell traction force [46, 47].

In the canonical integrin–talin–actin mechanotransduction pathway [4, 48, 49, 50, 51, 52], strengthening of the ECM–integrin linkage induces conformational changes in integrins, leading to mechanosensitive recruitment of talin from the cytosol [4, 28, 52, 53]. Talin bridges integrins to F‐actin filaments, forming a physical connection between the ECM and the cytoskeleton. Under mechanical tension, talin rod domains unfold, exposing additional binding sites for vinculin [52], which reinforces the actin linkage [49] and stabilizes the adhesion. Our study supports this model: applying force to integrins led to increased focal adhesion area and talin recruitment, promoting talin unfolding and subsequent F‐actin reinforcement.

Previous mechanobiology studies highlight the reversible nature of cellular responses to stiffness, demonstrating a positive and reversible correlation with talin intensity and F‐actin levels [4, 5, 54]. We assessed the potential reversibility of force responses in relation to talin intensity, F‐actin, and cell traction force. Our findings indicated that both talin intensity and F‐actin levels returned to their baseline values following the termination of integrin force application, at a rate similar to their initial increase. In contrast, cell traction force demonstrated only a partial recovery. This observation implies the reversibility of force application techniques as well as cellular responses in relation to talin, F‐actin, and traction force. The incomplete recovery of traction force may be attributed to the involvement of additional cellular components that influence the determination of cell traction force.

In conclusion, we have expanded the motor‐based actuation surfaces [19] to soft hydrogels and validated additional functionalities. When combined with traction force studies, the motor/hydrogel allows investigation of cellular responses to integrin actuation, like talin recruitment, F‐actin polymerization, or cell forces, as well as their potential reversibility. Similar studies with other FA proteins will help to elucidate the dynamic interplay of proteins and cytoskeletal structures at the FA for mechanotransduction. Future development of the platform to integrate DNA force sensors might enable in situ quantification of the transmitted force to the FAs during photoactuation.

## 4 Experimental Section

### 4.1 Preparation of P(AAm‐AA) Hydrogel

P(AAm‐AA) hydrogel thin films were prepared by adapting a published protocol (Farrukh et al., 2017){Farrukh, 2017 #95}. Acrylamide (8 wt.%, Sigma, A9099) and N, N‐methylene‐bis‐acrylamide (0.264 wt.%, Sigma, 1015460100) comonomers were dissolved in 5 mL PBS (pH7). Acrylic acid (AA) (2% to acrylamide, Sigma, 147230) was added to the mixture. This comonomer concentration targeted P(AAm‐AA) hydrogels with a Young's Modulus of 19.6 kPa [55]. The pH of the comonomer solution was adjusted to pH 8 by using 0.1 m NaOH aq. solution. The solution was flushed with N_2_ gas to displace O_2_ and avoid inhibition of the free radical polymerization. The initiator ammonium persulfate (10% solution, 1/100 of total volume, Sigma A3678) and TEMED (Sigma, T9281) catalyst (1/1000 of total volume) were added to the mixture. Thin films were prepared between two glass plates (see thin film preparation for TFM below). For screening experiments to optimize hydrogel preparation, 300 µL of monomer solution were polymerized in a 6‐well plate.

### 4.2 Functionalization of the P(AAm‐AA) Hydrogels with DBCO‐NH_2_

For screening experiments, P(AAm‐AA) hydrogel was incubated with 1 mL solution of 0.4 m N‐(3‐(dimethylamino) propyl)‐N′ ethylcarbodiimide hydrochloride (EDC, Sigma, 8009078500), 0.1 m N‐hydroxysuccinimide (NHS, Sigma, 130672) in 2‐(N‐morpho)‐ethanesulfonic acid buffer (prepared by mixing equal molar solution of 0.1 m 2‐(N‐morpholino) ethanesulfonic acid buffer (MES, Thermofischer J62231.AP) and 0.5 m NaCl) for 15 min, then three time wash with PBS followed by incubation with 1 mL of DBCO‐NH_2_ (Sigma, 761540) at concentrations between 0.05 and 5 mm in Dimethyl sulfoxide for 1 h. The hydrogel was washed with PBS three times.

### 4.3 Coupling of Motor/PEG Conjugate to DBCO Functionalized Hydrogel

The motor/PEG conjugate was coupled to the DBCO‐ P(AAm) hydrogel by the strain‐promoted azide‐alkyne cycloaddition reaction. The DBCO‐hydrogel was incubated overnight at 4°C with concentrations between 0.1 and 1.5 mg/mL of the motor/PEG conjugate solution in water (pH 7). On the next day, the hydrogel was washed thrice with PBS to remove unreacted motor/PEG conjugate. Unreacted DBCO group was quenched by incubating motor/PEG hydrogel with 1 µg/mL of O‐(2‐Azidoethyl)‐O′‐methyl‐triethylene glycol (PEG‐azide, Sigma 712590) solution in water for 1 h at RT followed by washing trice with PBS.

### 4.4 Coupling of RGD to the Motor/PEG Conjugate Functionalized Hydrogel

The azide group of peptides cyclo (RGDfK‐N_3_) (Thermofisher, 17293766), where the Lysine rest was substituted by 6‐azido‐L‐Lysine, was coupled to the free alkyne terminal of motor/PEG hydrogel by copper‐catalyzed azide–alkyne cycloaddition. The motor/PEG hydrogel was incubated with 100 µL of the 0.01–1 mg/mL peptide solution in PBS (pH7) containing CuSO_4_·5H_2_O (10 µL, 0.03 mg/mL, 0.125 mm, Sigma C8027), tris(3‐hydroxypropyltriazolylmethyl) amine (10 µL, 0.271 mg/mL, 0.62 mm, Sigma, 762342) and sodium ascorbate (10 µL, 0.495 mg/mL, 2.5 mm, Sigma, A4034) for 90 min at RT. After reaction, the motor/PEG/RGD hydrogel was rinsed three times with PBS to remove unreacted compound, and motor/PEG/RGD conjugated hydrogel surface was used immediately for cell seeding. All reagents used in these methods were prepared fresh from solid stocks unless mentioned otherwise.

### 4.5 Characterization of the Coupling Reactions to the Hydrogel by UV Spectroscopy

For all screening experiments, polymerized hydrogels were filled in quartz cuvettes to perform UV spectroscopy. The coupling of azadibenzocyclooctyne‐amine (DBCO‐amine) and motor/PEG conjugate to the P(AAm‐AA) hydrogel were monitored by UV spectroscopy measurements in a cuvette filled with PBS. The coupling of DBCO‐amine to the hydrogel was quantified by the increase in absorbance at 310 nm. The absorbance spectrum of the pure motor/PEG conjugate solution was recorded from 200–800 nm. The coupling of the motor/PEG conjugate was quantified by the increase in absorbance at 340 nm and the decrease in absorbance at 310 nm.

For the quantification of potential UV‐induced temperature changes, motor/PEG‐conjugated hydrogels were prepared following the same protocol used for actuation and traction force measurements. 10 µL of a co‐monomer solution containing temperature‐insensitive fluorescent beads (FluoSpheres, 0.2 µm, Dark Red, Thermo Fisher Scientific, F8807; 1:1000 dilution, 3.4 × 10⁹ particles/mL) was used for each sample. The hydrogel was functionalized with Amin‐DBCO via EDC/NHS‐mediated coupling, followed by strain‐promoted azide–alkyne cycloaddition with the motor/PEG conjugate. The medium was supplemented with 0.5 µm Rhodamine B (Rhb) [56]. The temperature of the system was controlled by the environmental chamber.

To obtain the calibration curve, Rhb fluorescence was measured as a function of sample temperature while varying the medium temperature between 26°C and 36°C. Higher temperatures could not be reached due to the heating limitations of the Okolab system on the existing microscope setup. However, Rhb fluorescence was known to exhibit a linear dependence on temperature within the 0–80°C range and, therefore, fluorescence values for higher temperatures could be extrapolated from the experimental calibration curve [56]. Embedded dark red beads served as temperature‐insensitive fiducial markers for surface localization during motor activation. The slope of Rhb fluorescence intensity vs. temperature was determined from a calibration curve and subsequently used to estimate local temperature variations within illuminated ROIs under different laser power densities.

Imaging was performed under the same conditions as the mechano‐actuation experiments using a Nikon microscope (100×/1.45 NA objective) equipped with an Andor iXon Ultra EMCCD camera, a 375 nm Opti‐Microscan point‐scanning device (Acal BFi), and an Okolab on‐stage incubator (±0.3°C). Rhb fluorescence was recorded using a 561 nm laser, while the dark‐red beads were imaged using a 638 nm laser. The bulk hydrogel temperature was monitored in real time with a JUMO iTRON 32 probe (±0.2°C).

### 4.6 Preparation of Motor/PEG/RGD Conjugate Hydrogels Surface for Single Cell Traction Force and Opto‐Actuation

For opto‐actuation and cell traction force experiments, a drop of 10 µL of co‐monomer solution containing fluorescent beads (10 µL/mL of Carboxylate‐Modified MicroSpheres coated with red fluorescent dye, diameter 0.2 µm, Thermofisher, F8801) was placed on a Sigma coated glass coverslip (diameter, 13 mm) and covered with a 3‐acryloxypropyl‐trimethoxysilane (Sigma, 440140) functionalized glass slide (diameter 35 mm, ibidi, 81158) and left to rest for 10 min. During this time, the beads sedimented and concentrated at the Sigma‐coated cover glass—gel interface due to gravity and slow gel polymerization rate. Once polymerization finished, the sandwiched P(AAm‐AA) hydrogel was soaked in PBS for 30 min, and the Sigmacoated coverslips were peeled off. With this protocol, swollen hydrogels with a thickness of 100 ± 10 µm were fabricated.

The glass‐supported P(AAm‐AA) thin film was functionalized with amine‐DBCO after EDC/NHS activation by incubating with a 100 µL solution of 1 mm amine‐DBCO solution in DMSO for 1 h at room temperature. The hydrogel was washed three times with PBS to remove unreacted amine‐DBCO.

The motor/PEG conjugate was coupled to the hydrogel‐DBCO by incubating overnight at 4°C with a 1 mg/mL solution of the motor/PEG conjugate in water. Rinse three times to eliminate any unreacted compounds. The remaining functional DBCO groups in the hydrogel were quenched by a 1 h incubation with a 20 mm in water solution of O‐(2‐Azidoethyl)‐O’‐methyl‐triethylene glycol (PEG‐azide). Finally, the hydrogel was incubated with a 0.1 mg/mL solution of cyclo (RGDfK‐N_3_), rinsed three times with PBS to remove unreacted compound, and the motor/PEG/RGD hydrogel was used immediately for cell seeding.

All reagents used in these methods were prepared fresh from solid stocks. Remaining reaction conditions were the same as screening, unless mentioned otherwise.

### 4.7 Cell Culture

The talin1^−/−^ mouse embryonic kidney (MEK) fibroblasts stably re‐expressing talin1‐YPet cells were provided by Prof C. Grashoff (University Munster) [57]. Cells were cultured in high‐glucose Dulbecco's Modified Eagle Medium (DMEM, Gibco, Thermofisher, 11965092) supplemented with 10% fetal bovine serum (Gibco, Thermofisher,10270106) and 1% Penstrep (P/S, Thermofisher 15140122). For imaging experiments, we used DMEM without phenol red (Thermofisher, 21063029) containing high glucose, HEPES, and glutamine supplemented with 10% FBS and 1X Penstrep. A total of 70 000 cells were seeded onto a 35‐mm glass‐bottom dish containing a cRGD/motor/PEG‐functionalized hydrogel (13‐mm diameter, 100‐µm thickness) in 2 mL phenol red–free culture medium. Cells attach to the upper surface of the functionalized hydrogel, and after 10 min, unattached cells were removed by gently washing with fresh medium. Cultures were then maintained for 2 h in a CO_2_ incubator prior to experiments. For the Y27 condition, the culture media was supplemented with 5 µm ROCK inhibitor (Y27632, Sigma, Y0503). Inhibitor was added to fresh culture media and maintained throughout the experimental duration.

### 4.8 Labeling F‐actin

For labeling F‐actin, talin1‐YPet fibroblast were seeded on the motor/PEG/RGD hydrogel and immediately incubated with 1X probe solution of SPY650‐FastAct (Spirochrome, SC505).

### 4.9 Microscopy Imaging

Microscopy was performed on an Eclipse Ti‐E motorized inverted total internal reflection fluorescence (TIRF) microscope (Nikon Instruments, Melville, NY, USA) equipped with on stage incubator (Okolab USA Inc, San Bruno, CA, USA) to maintain 5% CO_2_ and 37°C temperature and humidity. Perfect‐Focus system (PFS) was used for keeping cells in focus. This microscope was equipped with 488, 561 and 638 nm lasers and a standard cube.

Imaging was done with a special objective Plan Apochromat Lambda 100X DIC N2 (MRD01905), which has a transmittance in 375 nm and equipped with Andor iXon Ultra EMCCD 16‐bit Camera (DU‐888U3‐CS0‐#BV) with 1024×1024 pixels (13×13 µm pixel size) controlled with NIS‐Element software. Cells stable expressing Talin‐YPet was captured with 488 lasers,1s exposure, 300 EM gain, and 1% laser power. Cells labelled with SPY650‐FastAct or Far‐Red bead with 638 lasers with 100 ms exposure, No‐ EM gain, and 0.2% laser power, while Actin RFP or Red bead with 561 nm lasers with 100 ms exposure 0.2% laser power. All imaging was done in epifluorescence mode.

To get the bead position when the hydrogel was not deformed by the cell, after finishing imaging, cells were removed with TrypLE express (Gibco, Thermofisher, 12604013). The image of bead particles on the un‐deformation gel substrate were acquired in the areas where cells were imaged beforehand.

### 4.10 Motor Illumination Condition

A UV Opti‐Microscan point scanning device (Acal BFi) equipped with a 375 nm (70 mW, Cobalt Rumba) laser was incorporated into the upper deck of a Nikon TIRF microscope. Prior to each experiment, the calibration of laser drift and focus was performed using a chromium calibration slide. The hydrogel surface was scanned sequentially utilizing two custom circular ROIs positioned diagonally, each with a diameter of 7 µm, within the NIS‐Element Opti‐scan software. Each ROI was scanned for a total duration of 6 s, with a pixel dwell time of 1800 microseconds, maintained consistently at 20‐s intervals during imaging. A 2‐min image without exposure was captured before the activation of the motor/PEG conjugate through UV mediation.

### 4.11 Quantification of Cell Morphometric Parameters

The spread area, perimeter, circularity, and aspect ratio of MEK cells were quantified from talin fluorescence images using Fiji/ImageJ (NIH, USA). Rounded cells were selected for mechano‐actuation experiments and subsequent analysis. Individual cells were manually segmented by drawing regions of interest (ROIs) around their boundaries based on the talin fluorescence signal. From each ROI, the following parameters were extracted using the Measure function: cell spread area (µm^2^), perimeter (µm), circularity (4𝜋×area/perimeter^2^), and aspect ratio (major axis/minor axis of the best‐fit ellipse).

### 4.12 Traction Force Reconstruction

Deformed and relaxed bead movies obtained during experiments were aligned at a subpixel level to mitigate any drift, utilizing the Align Slice tool, an ImageJ plugin [58]. The reconstruction of the cell traction map was performed using publicly available MATLAB‐based cTFM Package software (https://github.com/DanuserLab/u‐inferforce) [9]. Briefly, the displacement field map of the movie was derived through a cross‐correlation‐based particle tracking velocimetry method, which facilitates the tracking of local displacements, with a maximum observed displacement of 0.7 µm (1 pixel = 130 nm) [59]. The calculation of cell traction force maps was executed from the displacement data using the Fourier transform traction cytometry method, applying a regularization value of 1 × 10^−6^ [60].

### 4.13 Segmentation and Tracking of Focal Adhesion, and Data Analysis

Talin‐YPet time‐lapse image stacks were employed for the segmentation of talin focal adhesions, which were subsequently overlaid onto the original talin, cell traction force, and F‐actin stacks to extract the corresponding values for talin, traction force, and F‐actin. The original Talin‐YPet images underwent preprocessing that included denoising using Nikon denoise AI, histogram‐based bleach correction, and application of a Mexican hat filter with a 3 × 3 × 3 voxel kernel. An adaptive thresholding technique was then applied, calibrated to correspond with the manually calculated size of the focal adhesions. This threshold value was consistently utilized across the entire stack to generate a segmented binary focal adhesion stack (Figure S4a,b).

An image stack comprising three channels—binary focal adhesion, raw talin intensity, and cell traction force magnitude or F‐actin intensity—was generated using the merge channel function in Fiji. The resulting three‐channel time‐lapse stack underwent processing with the TrackMate plugin in Fiji [61, 62] to measure the trajectories of focal adhesion puncta, ensuring the unmixing and consistent tracking of the same location throughout the time‐lapse sequence. Subsequently, the TrackMate plugin was activated, where illuminated areas were selected, and a label image detector algorithm was chosen. Parameters, including initial thresholding, were configured to guarantee precise detection of focal adhesion spots. Upon detection, the LAP tracker [63] inking algorithm was employed, utilizing a frame‐to‐frame linking distance of 2.6 µm, a track gap‐closing distance of 2.6 µm, and a maximum frame gap of 3 to establish trajectories over time. Newly appearing or disappearing focal spots during the frames were filtered out. Following the tracking process, the software facilitated quantitative analyses, including ROI of the spot, mean values of talin, traction force, F‐actin intensity, and trajectory persistence. These results were subsequently exported to Origin 9.1 for visualization and statistical evaluation.

All intensity quantification was conducted using raw images. The intensity values for talin, traction force, and F‐actin underwent denoising through smoothing via Fast Fourier Transform (FFT), employing a cutoff value of four in Origin 9.1. The mean and standard deviation data were reported in all plots.

### 4.14 Statistical Analysis

All data were reported as average ±standard deviation (mean±s.d.). All analyses were performed using Origin9.1 (OriginLab Software) and Prizm (Graphpad Software), and a *p* value <0.05 was considered significant. Detailed statistical parameters was provided additionally in an excel sheet format.

## Conflicts of Interest

The authors declare no conflicts of interest.

## Supporting information




**Supporting File 1**: advs73445‐sup‐0001‐SuppMat.pdf.

**Supporting File 2**: advs73445‐sup‐0002‐MovieS1.avi.

**Supporting File 3**: advs73445‐sup‐0003‐MovieS2.avi.

**Supporting File 4**: advs73445‐sup‐0004‐MovieS3.avi.

**Supporting File 5**: advs73445‐sup‐0005‐MovieS4.avi.

**Supporting File 6**: advs73445‐sup‐0006‐MovieS5.avi.

**Supporting File 7**: advs73445‐sup‐0007‐MovieS6.avi.

**Supporting File 8**: advs73445‐sup‐0008‐MovieS7.avi.

## Data Availability

The data that support the findings of this study are available from the corresponding author upon reasonable request.
