# Supplementary material for: An Opto‐Actuated Hydrogel for Cell Mechanoactuation and Real‐Time Force Monitoring
Source: Adv Sci (Weinh). 2026 Jan 4;13(10):e11538. doi: 10.1002/advs.202511538 (PMC12915213; doi:10.1002/advs.202511538)
Supplement: Supplementary file 1 — Supporting File 1: advs73445‐sup‐0001‐SuppMat.pdf. [file ADVS-13-e11538-s006.pdf]

Supporting material

An opto-actuated hydrogel for cell mechanoactuation and real-time force monitoring

Rinku Kumar1, Marc A. Fernandez-Yague1,2, Adrien Bessaguet5,6, Hosoowi Lee5,6, Nicolas Giuseppone5,6, Andrés J. García7,8, Aránzazu del Campo1,3✉

Figure s1……………………………………………………………………02
Figure s2……………………………………………………………………03
Figure s3……………………………………………………………………04
Figure s4……………………………………………………………………05
Figure s5……………………………………………………………………06
Figure s6……………………………………………………………………07
Figure s7……………………………………………………………………10
Figure s8……………………………………………………………………11
Figure s9……………………………………………………………………13
Figure s10……………………………………………………………………14
Figure s11……………………………………………………………………15
Figure s12……………………………………………………………………16
Figure s13……………………………………………………………………18
Figure s14……………………………………………………………………19

Figure S1

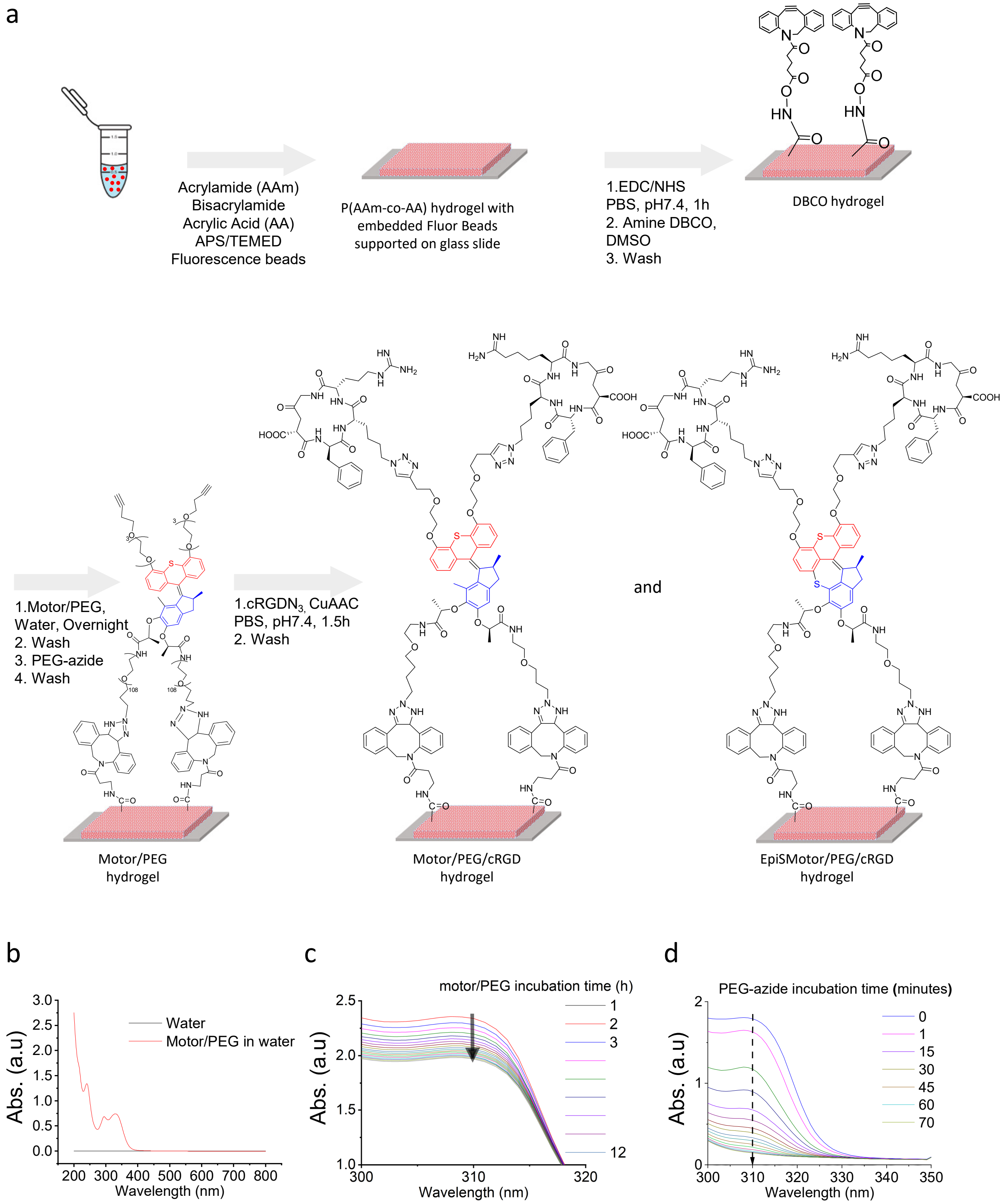

Figure s1: Schematic of synthesis of cRGD motor functionalized hydrogel and their charterization. a) Schematic of chemical rection followed for the functionalization of PAA surface with motor and EpiSMotor. **b)** UV spectra (200-800 nm) of the motor/PEG solution (0.25 mg/ml) in water. **c)** UV spectra (300-320 nm) of the DBCO-functionalized hydrogel after incubation with 1 mg/mL motor/PEG conjugate for increasing incubation time. **d)** UV spectra of DBCO-functionalized hydrogels incubated with 1 µl/mL PEG-azide for increasing times. The formation of the triazole group in a) and c) leads to a decay in absorbance at 310 nm.

Figure S2

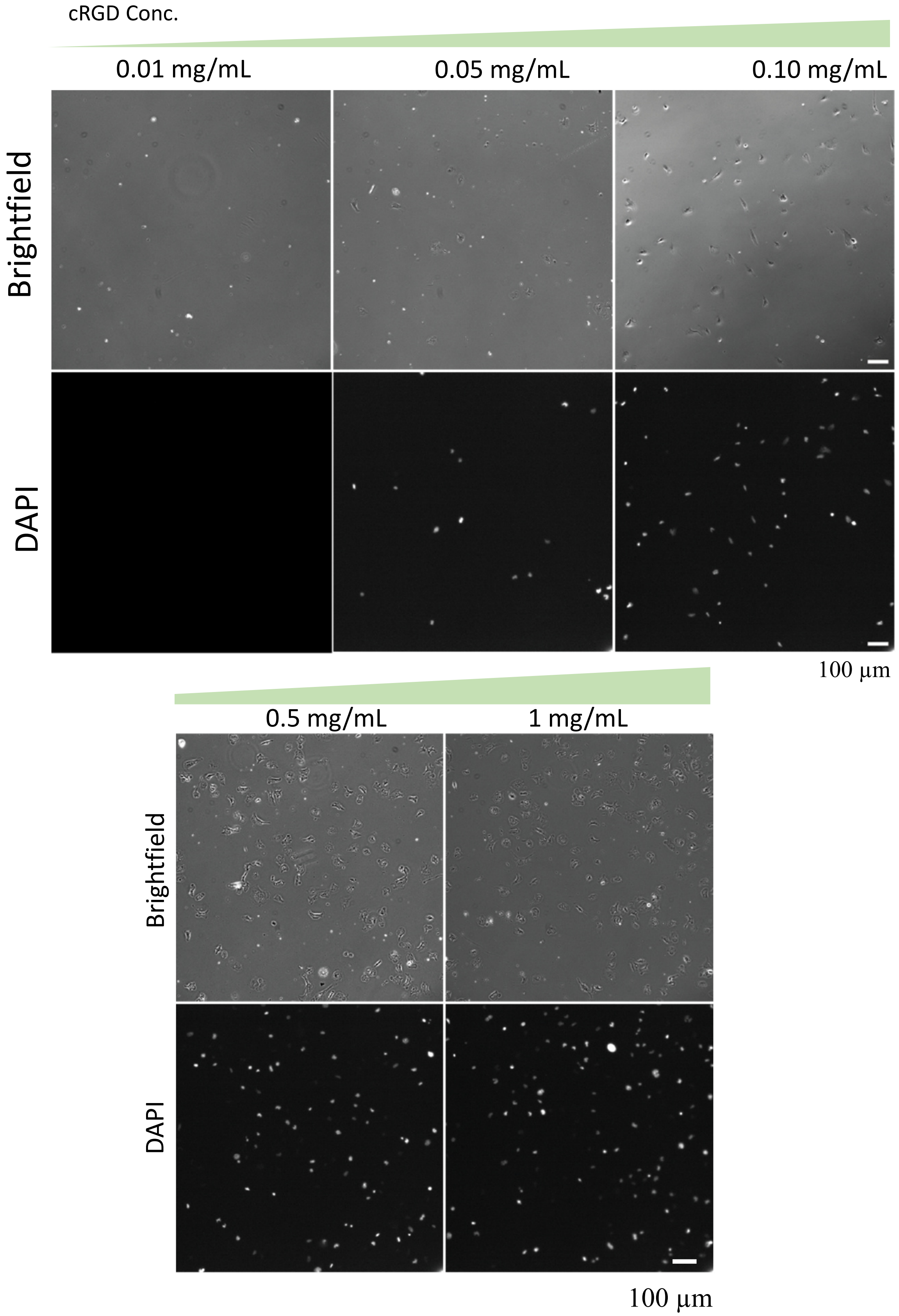

Figure s2: Cells grow specifically on motor/PEG/RGD in a concentration-dependent manner. Brightfield and DAPI images of Talin1-yPet expressing mouse embryonic kidney (MEK) cells at seeding density of $2.2x10^4$ cells/cm^2 were incubated with the motor/PEG/RGD functionalized hydrogels at different RGD incubation concentrations (0.01-1 mg/mL) for 1.5 hours.

Figure S3

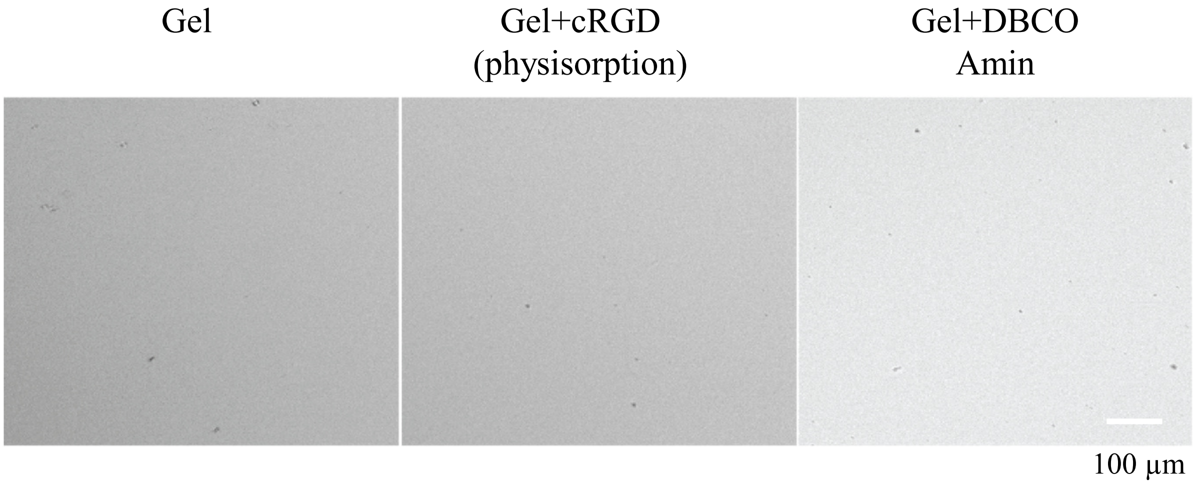

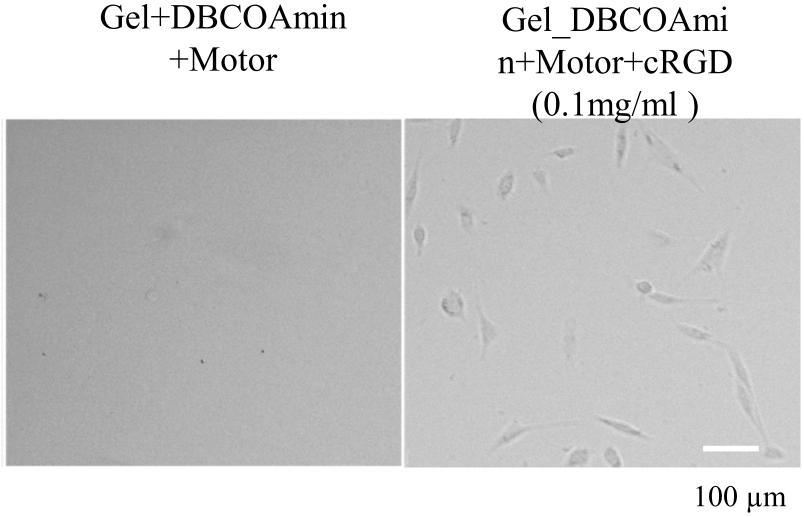

Figure s3: Cells grow specifically on motor/PEG/cRGD. No cell spreading was observed on either gel or hydrogels modified with gel/cRGD, gel/AminDBCO and gel/AminDBCO/motor/PEG conjugate. PAA hydrogel functionalized with at least 0.1 mg/ml of cRGD, gel/AminDBCO/motor/PEG/RGD.

Figure S4

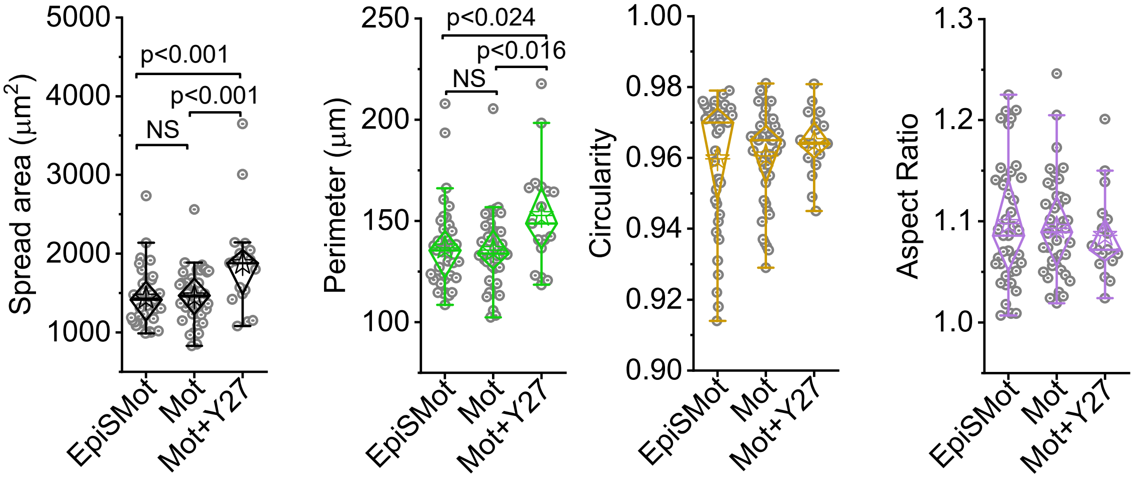

Figure s4: Dimensional analysis of MEK cells used in experiments. Spread area, perimeter, circularity and aspect ratio of MEK cells selected for mechano-actuation studies on EpiSMot/PEG RGD hydrogel, Motor/PEG RGD hydrogel and Motor/PEG RGD hydrogel subjected to a 1-hour pre-treatment with 5 μM Y27632. Measurements were taken prior to mechano-actuation for the EpiSMot and Mot conditions, while for the Mot+Y27 condition, the analysis was performed after the 1-hour pre-treatment with Y27632. The spread area and perimeter showed a significant difference among the three conditions (EpiSMot, Mot, and Mot+Y27), whereas the circularity and aspect ratio did not differ significantly between conditions. Data include all experiments performed under continuous and cyclic mechano-actuation, as well as talin, traction force and F-actin assays. Total number of analyzed cells: n = 41, 42, and 17 for EpiSMot, Mot, and Mot+Y27, respectively.

Statistical analysis was performed by one-way ANOVA with Dunn's multiple comparison test: **spread area (***EpiSMot vs. Mot, p=0.9797, EpiSMot vs. Mot+Y27, p>0.0012, Mot vs. Mot+Y27, p>0.0019);* **perimeter (***EpiSMot vs. Mot, p=0.9678, EpiSMot vs. Mot+Y27, p>0.0024, Mot vs. Mot+Y27, p>0.0014)* .

Mean± s.d. for spread area (*EpiSMot* =1435±359; *Mot*=1453±341; *Mot+Y27*=1874±656). **perimeter** (*EpiSMot* =137±21; *Mot* =134±13; *Mot+Y27* =152±27). **circularity (***EpiSMot*=0.96±0.02; *Mot* =0.96±0.01; *Mot+Y27* =0.97±0.01). **aspect ratio (***EpiSMot*=1.09±0.06; *Mot* =1.09±0.05; *Mot+Y27* =1.09±0.05).

Figure S5

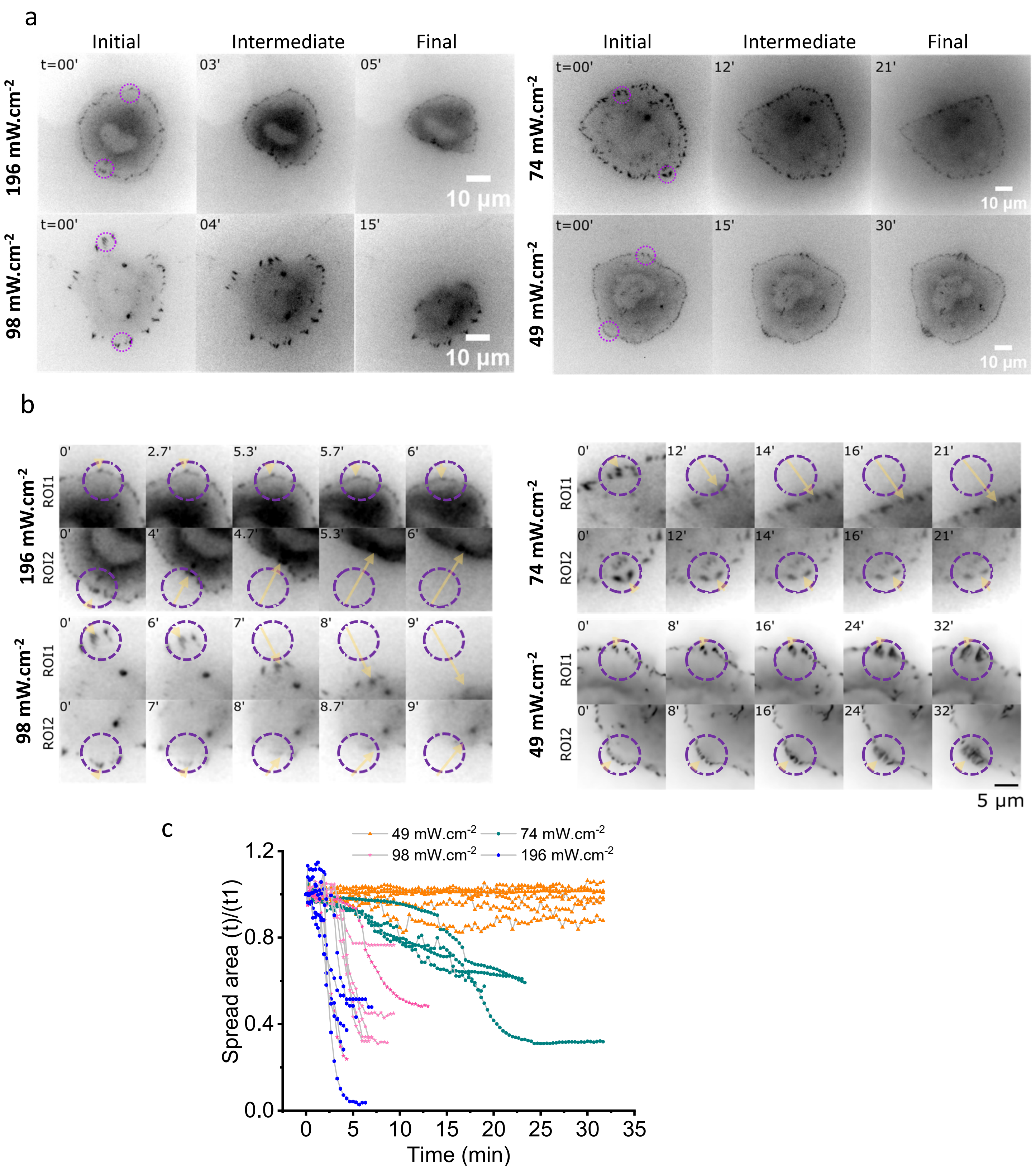

Figure s5: Optimization of the irradiation dose of 375 nm laser illumination of cells for motor activation. a Talin-yPet cells expressing MEK cells were cultured on a motor-functionalized hydrogel surface and exposed to different power density of UV light (pixel residence time: 1800 µs). Dissociation of focal adhesion was observed at a UV light power density above 49 mW.cm^{-2}.Three stage of dissociation at different time points for different condition is shown. **b** Close-up images of Fig.s3a showing the dissociation of focal adhesions at different stages. The arrow shows the position from initial to final retraction as observed during the experiments. **c** Total cell **s**pread area change followed for different illumination conditions.

Figure S6

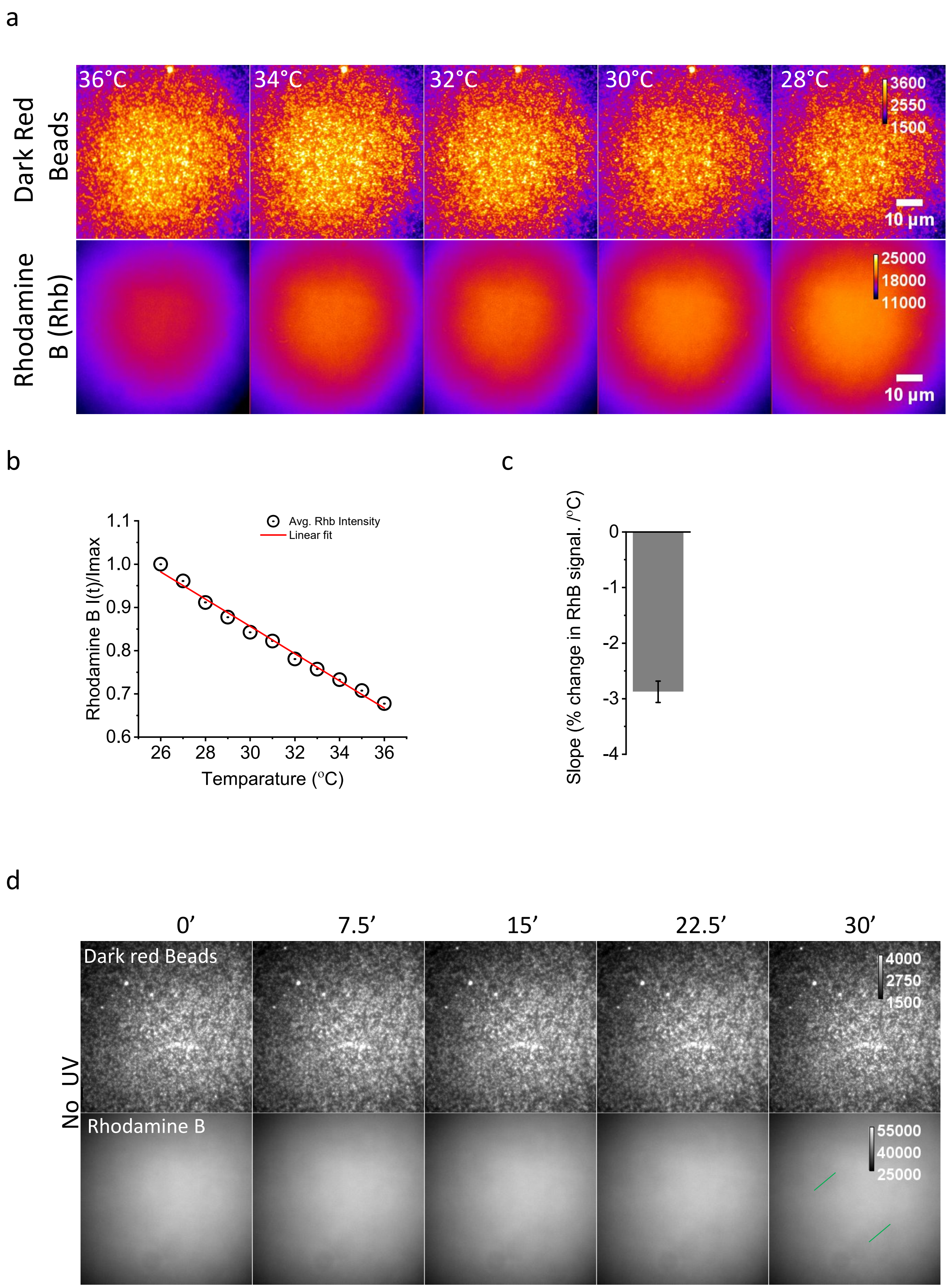
a
36°C
34°C
32°C
30°C
28°C
3600
2550
1500
10 µm
Dark Red Beads
Rhodamine B (Rhb)
25000
18000
11000
10 µm
b
Avg. Rhb Intensity
Linear fit
Rhodamine B I(t)/Imax
Temparature (°C)
c
Slope (% change in RhB signal. /°C)
d
0'
7.5'
15'
22.5'
30'
Dark red Beads
Rhodamine B
No UV
4000
2750
1500
55000
40000
25000

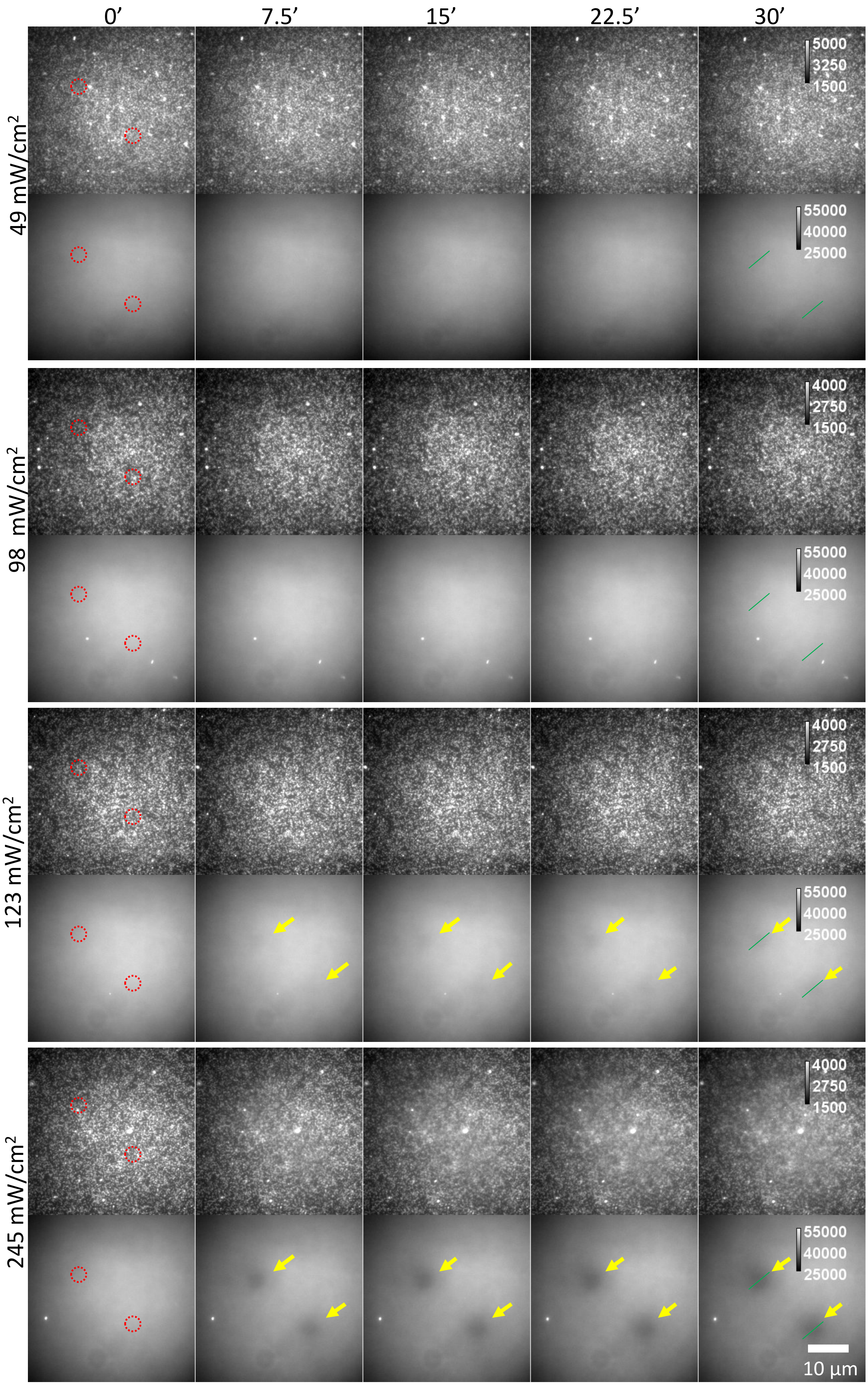
0'
7.5'
15'
22.5'
30'
49 mW/cm2
98 mW/cm2
123 mW/cm2
245 mW/cm2
5000
3250
1500
55000
40000
25000
4000
2750
1500
10 µm

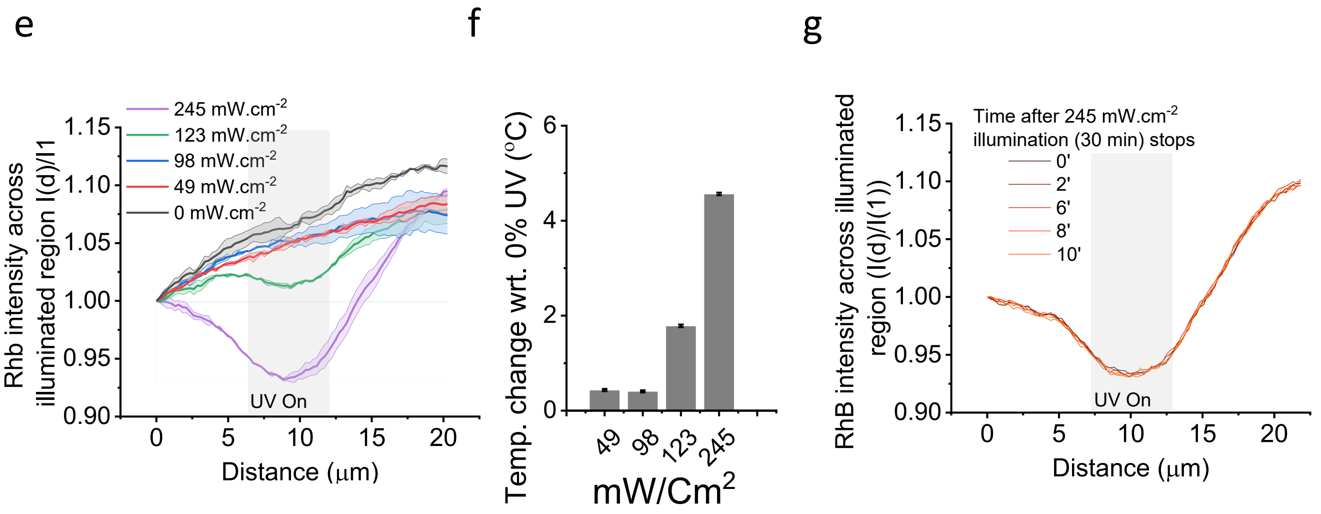

Figure s6: Quantification of potential UV-induced temperature changes at ROIs of motor/PEG conjugated hydrogels using Rhodamine B (Rhb) as temperature-sensitive dye. Motor/PEG/RGD-conjugated hydrogel films embedded with dark-red fluorescent beads and supplemented with Rhb containing medium were prepared as described in the Methods section. Under conditions identical to continuous opto-actuation experiments, the top motor/PEG surface was identified by focusing on the fluorescent bead layer. Two circular ROIs (7 µm diameter) on the hydrogel surface were illuminated using a 375 nm laser at varying power densities for 30 minutes. At defined time intervals, both Rhb and bead fluorescence images were recorded. Rhb fluorescence intensity profiles across the illuminated ROIs were extracted from line-scan analyses.

a) Top: Color-coded Rhb fluorescence images of the motor-functionalized hydrogel surface acquired at different temperatures, showing progressive intensity changes corresponding to temperature variations. Bottom: Representative images of dark-red fluorescent beads used to locate the top surface of the motor/PEG hydrogel. The absence of intensity changes confirms that the bead fluorescence and focal plane remain unaffected by temperature.

b) Calibration curve showing Rhb fluorescence intensity as a function of sample temperature (26–36 °C).

c) Mean ± s.d. of slope values derived from linear fits to calibration data across four independent samples.

d) Representative gray scale images of Rhb (top) and reference bead (bottom) at 36 °C during ROIs illumination under conditions similar to mechano-actuation experiments with increasing laser power density and exposure time.

e) Line plot showing mean ± s.d. of Rhb fluorescence intensity across the irradiated ROIs (indicated by the green line in panel d) after 30 min of UV illumination at different power densities and 36 °C medium temperature. Local minima from gaussian profile center were extracted, which were converted to temperature changes using the calibration slopes from panel c. The no UV illumination condition was used as a reference. Illumination at power densities <98 mW.cm^{-2} did not lead to significant temperature variations at the illuminated site

f) Plot of maximum local temperature change in illuminated ROIs as a function of laser power density. The power density used for mechano-actuation (49 mW cm^{-2}) produced no significant temperature increase, whereas power densities above 123 mW cm^{-2} resulted in ≥ 2 °C rises. This indicates that the laser intensity used for opto-actuation does not induce measurable thermal artifacts.

g) To exclude photo-bleaching, Rhb fluorescence was monitored for additional 10 min beyond the 30 min illumination under the highest power density (245 mW cm^{-2}) finishes. Since freely diffusing Rhb would recover rapidly if bleaching occurred, the steady fluorescence intensity (mean ± s.d.) observed across both illuminated ROIs confirms the absence of photobleaching under maximal laser power.

Figure S7

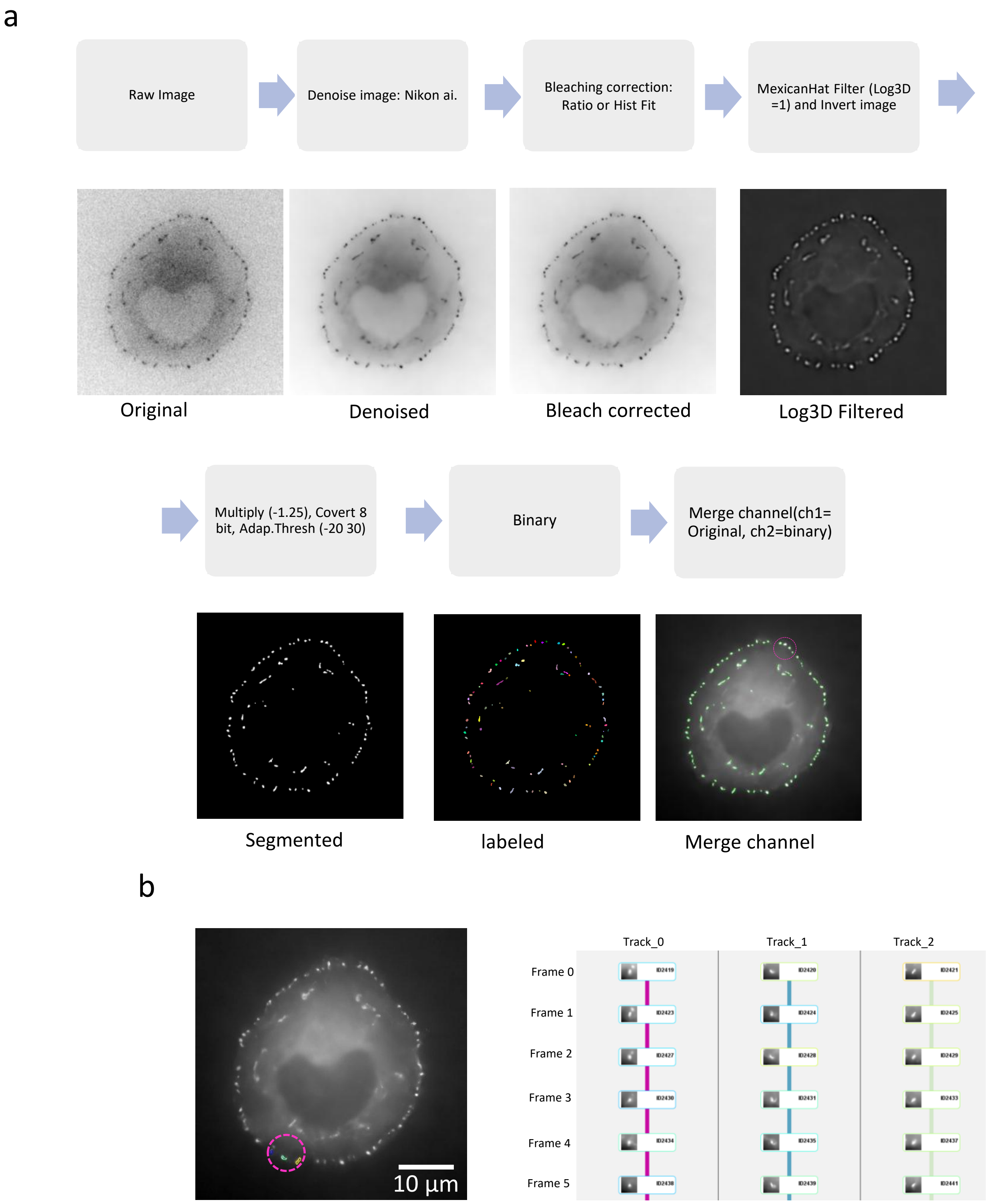

Figure s7: Workflow to segmentation and tracking of focal adhesion. a) Original Talin-yPet image was preprocessed by denoise with Nikon denoise ai, bleach corrected with histogram fit, maxican hat filter with 3x3x3 voxel kernel, adaptive threshold applied to create to binary image stack. Focal adhesion spot in ROI were detected using Trackmate7 plugin of Fiji, and tracked with LAP tracker linking algorithm to make sure same focal adhesion intensity and shape parameter is being followed in movie of f-actin, talin and cell traction force. **b)** Marked FAs of talin-yPet labeled cells grown on motor/PEG/RGD surface were illuminated with 375 nm laser inside two circle marked with pink dotted line. Individual focal adhesion within illuminated area were assigned a track and same track chased across time frame.

Figure S8

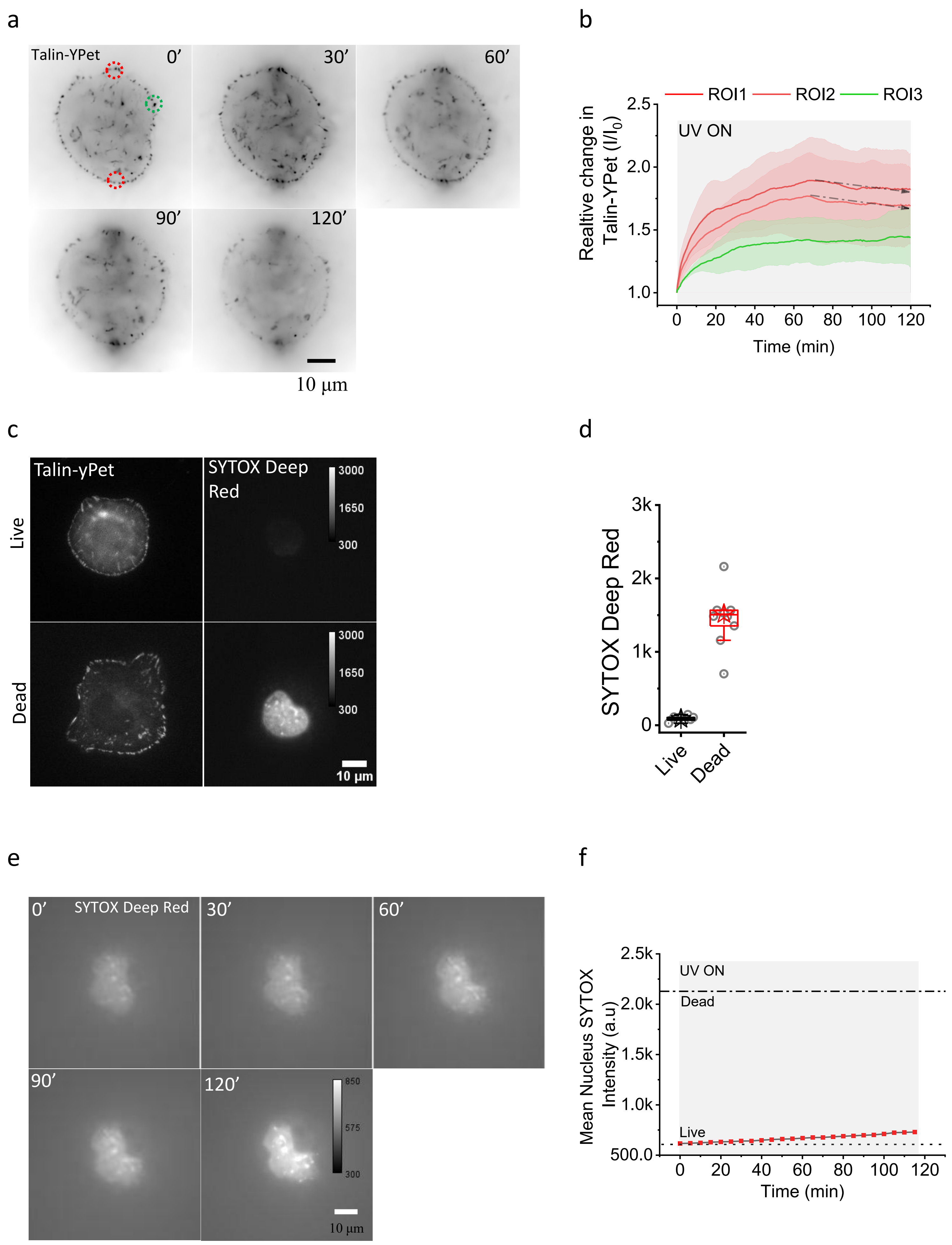
a
Talin-YPet
0'
30'
60'
90'
120'
10 μm
b
ROI1
ROI2
ROI3
UV ON
Realtive change in Talin-YPet (I/I_0)
2.5
2.0
1.5
1.0
0
20
40
60
80
100
120
Time (min)
c
Talin-yPet
SYTOX Deep Red
3000
1650
300
Live
Dead
10 μm
d
SYTOX Deep Red
3k
2k
1k
0
Live
Dead
e
0'
SYTOX Deep Red
30'
60'
90'
120'
850
575
300
10 μm
f
UV ON
Dead
Live
Mean Nucleus SYTOX Intensity (a.u)
2.5k
2.0k
1.5k
1.0k
500.0
0
20
40
60
80
100
120
Time (min)

Figure s8: Sustained mechano-actuation of talin and evaluation of cell vitality (membrane damage) in MEK fibroblasts. Prolonged focal adhesion mechano-actuation of talin-labeled cells was performed for over 30 min, following the same conditions as previous mechano-actuation experiments, but in the presence of the membrane-impermeable dye SYTOX Deep Red (0.5 µM, 30 min, Thermo Fisher, S11381) to simultaneously monitor long-term talin responses and potential cell membrane damage. For control conditions, live and ethanol-fixed (70%) MEK fibroblasts were incubated with SYTOX Deep Red (0.5 µM, 30 min), and fluorescence signals were acquired using the same dye concentration and imaging parameters as in the mechano-actuation experiments.

a) Representative images of Talin-YPet re-expressing MEK fibroblasts cultured on motor/PEG/RGD hydrogel interfaces during sustained mechano-actuation for 120 min. Illuminated ROIs are marked with red dotted circles, and the third reference ROI is indicated with green circles.

b) Line scatter plot (mean ± s.d.) showing average talin fluorescence intensity for each ROI (1–3) throughout the experiment. Data shown across two independent experiments.

c) Representative images of live Talin-YPet re-expressing MEK fibroblast and dead cells (fixed with 70% ethanol) incubated with the membrane-impermeable nuclear dye SYTOX Deep Red (0.5 µM, 30 minutes). The strong nuclear SYTOX signal in dead cells confirms dye uptake following loss of membrane integrity. The same control conditions were used for supporting cyclic mechno-actuation experiments shown in figure S13.

d) Box plot illustrating the basal SYTOX signal levels in live and dead cells (mean ± s.d., >10 cells per condition).

e) Representative fluorescence SYTOX images of MEK fibroblasts cultured on motor/PEG/RGD hydrogel interfaces during sustained mechano-actuation for 120 min. Corresponding talin-YPet images are shown in panel a.

f) Line scatter plot showing SYTOX fluorescence signal levels during continuous mechano-actuation in Talin-YPet re-expressing MEK fibroblasts cultured on motor/PEG/RGD hydrogel interfaces, compared with dead cells control signal.

Figure S9

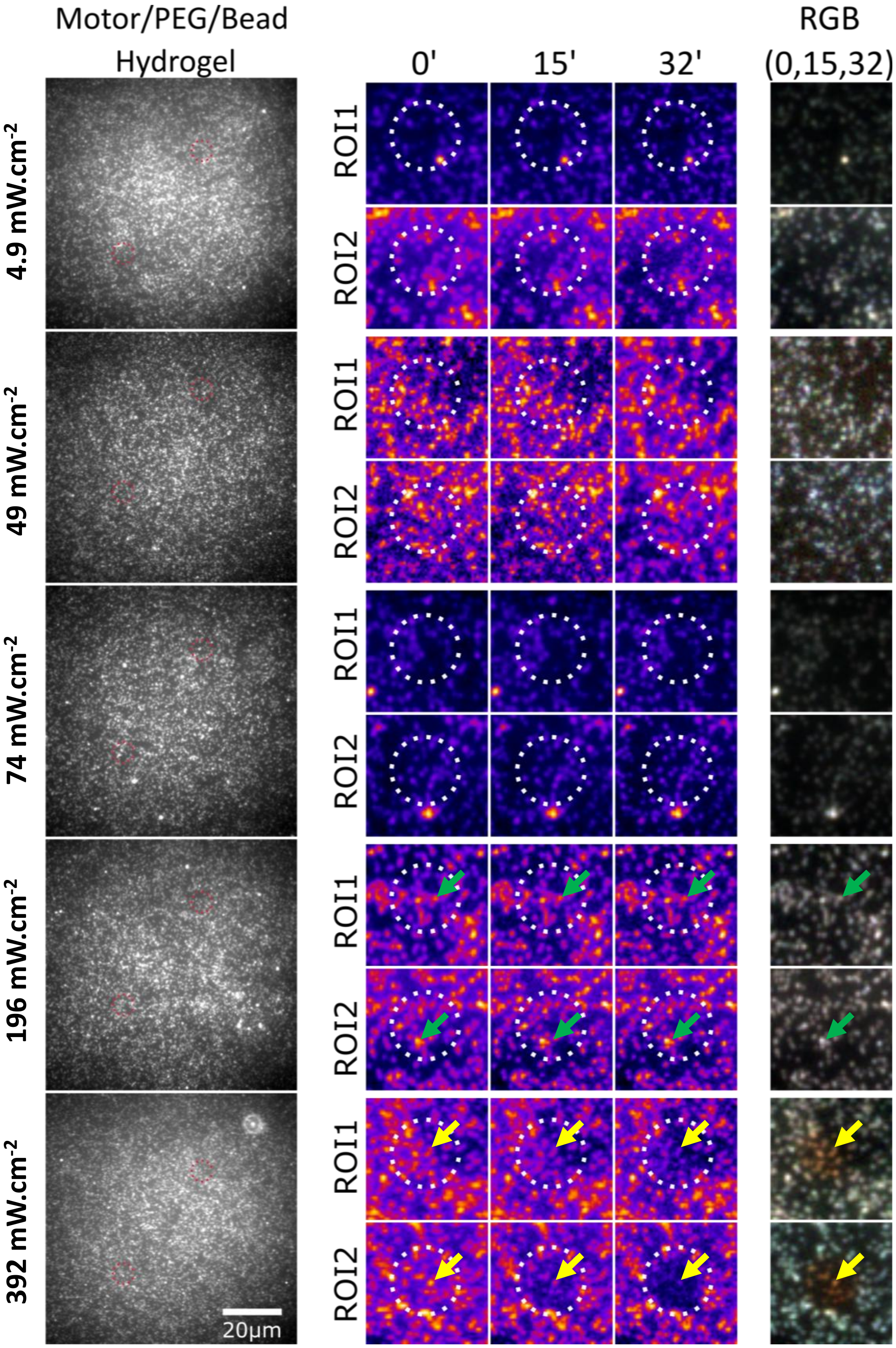

Figure s9: UV activation of motor functionalized hydrogel does not physically deform hydrogel. Motor/PEG/Bead hydrogel with carboxyl modified polystyrenes red fluorescence bead was exposed with different 375 nm laser density to check physical change in hydrogel. Images of Motor/PEG/Bead hydrogel, illuminated crop ROI images at 0, 15 and 32 minutes after UV exposure and merged RGB to show photobleaching. Higher laser intensity (> 196 mW.cm^{-2}) caused photo damage to the bead shown with yellow arrows. Green arrow show unbleached while yellow show bleaching event. For all experiments with used 49 mW.cm^{-2} laser power density.

Figure S10

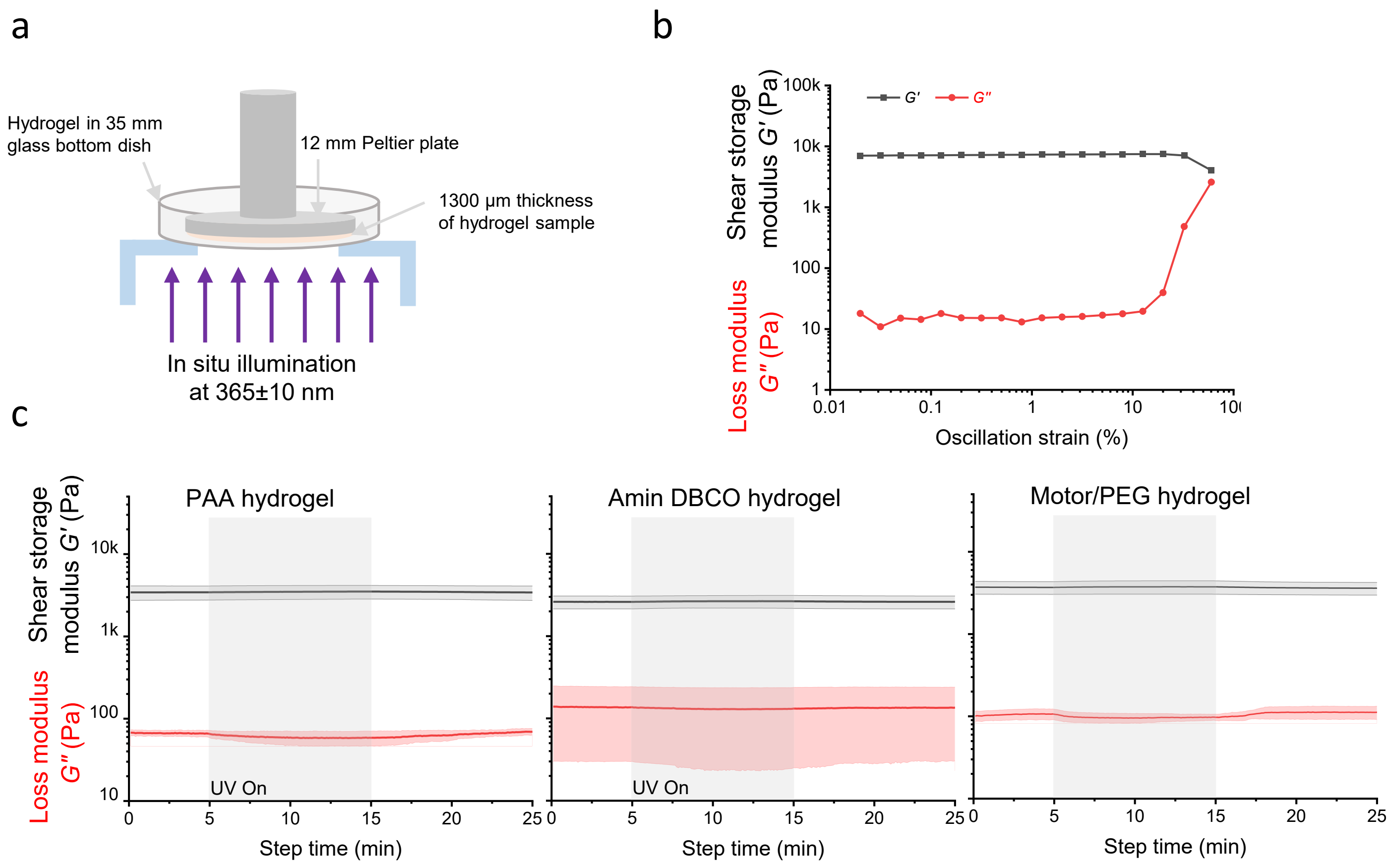

Figure s10: Rheological response of pristine, amin-DBCO and motor/PEG functionalized hydrogels upon UV illumination. The shear storage and loss modulus were measured using a stress-controlled rheometer (DHR 3, TA Instruments) under three reaction conditions. Polymerization was carried out in glass-bottom dishes, where the precursor solution was sandwiched between an upper 13 mm glass coverslip and dish surface. The synthesis conditions, including reactant concentrations and reaction time, were identical to those used in spectroscopic characterization. After polymerization and subsequent reactions, the hydrogel was mounted between the rheometer's 12 mm Peltier plate and bottom in situ UV illumination system (OmniCure, Series 1500, 365 nm, 29 mW cm^{-2}). Strain sweep test was performed on hydrogel polymerized directly between Peltier plate and the bottom quartz surface. The rheological response was recorded for five minutes without UV exposure, 10 minutes under the UV illumination, and 10 minutes after the UV light was turned off for all three experimental conditions.

a) Schematics of the plate-plate configuration used for rheological measurement showing experimental setup and hydrogel sample dimensions.

b) Strain sweep test of the pristine hydrogel polymerized between the rheometer plates, performed from 0.01% to 50% at a frequency of 1 Hz. The conditions 1% strain at 1 Hz fall within the linear viscoelastic region and were utilized for subsequent experiments.

c) Shear storage (G′) and loss moduli (G″) of pristine, amin-DBCO and motor/PEG functionalized hydrogels during UV exposure. Data are represented as a mean ± s.d from three independent sample, except for Amin DBCO which was tested with two replicates.

Figure S11

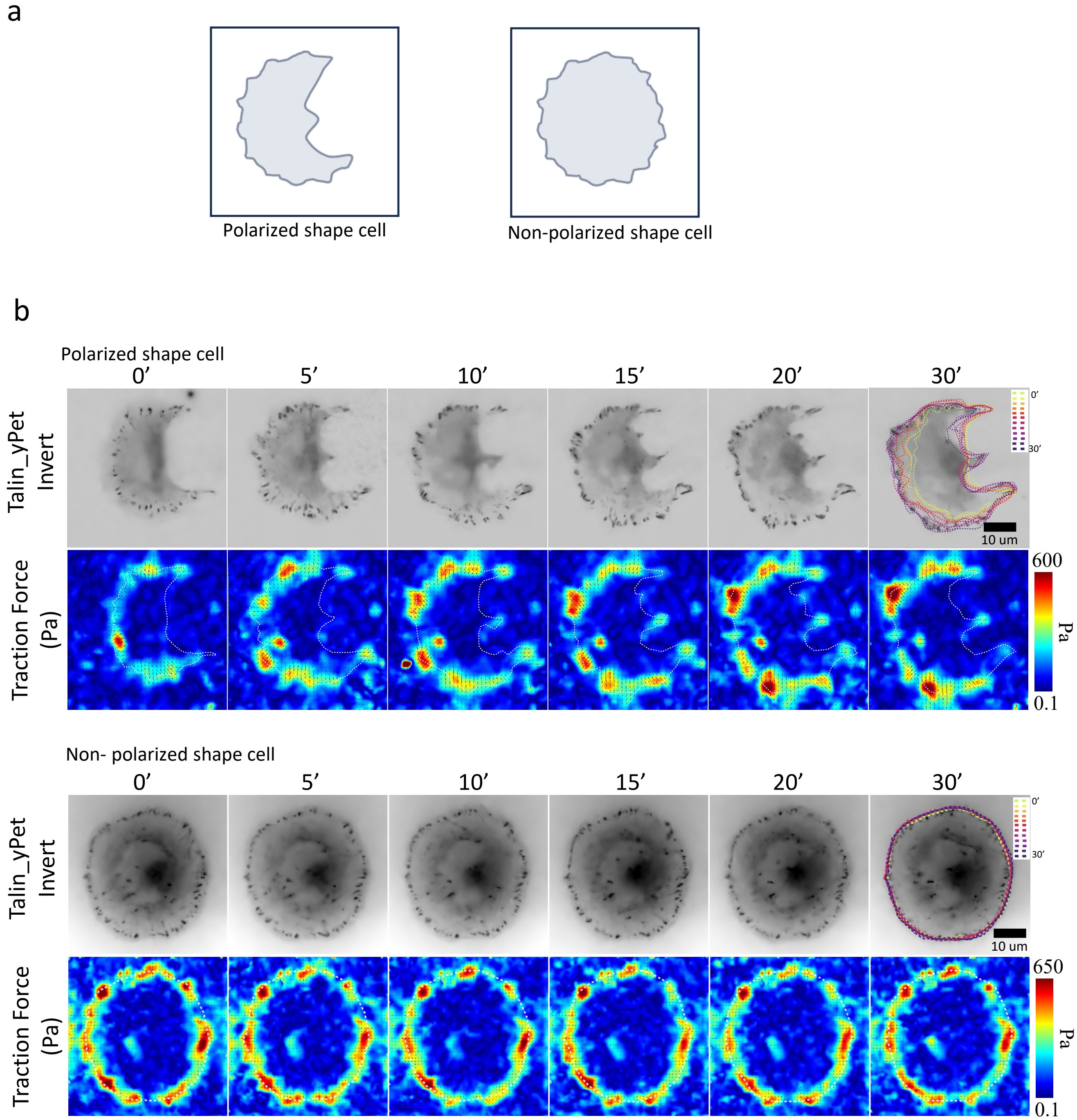

Figure s11: Validation of traction force measurement in polarized cells that exhibit migration and in non-polarized cells that show no movement. a) Diagram illustrating polarized and non polarized cells morphology. **b)** Time-lapse images of MEK cells expressing Talin-yPet cultured on a Motor/PEG/RGD hydrogel platform at different time points are shown above. Overlaid regions of cells at different time points show the migration of the cells during this period. The corresponding traction forces measured on the surface of the Motor/PEG/RGD hydrogel during cell migration indicate changes in traction force due to migration and validate the methods used to measure traction force. Similar data were presented for cells that have a rounded, non-polarized morphology and show non-significant movement, making them ideal for experiments that require long-term tracking of the same cells.

Figure S12

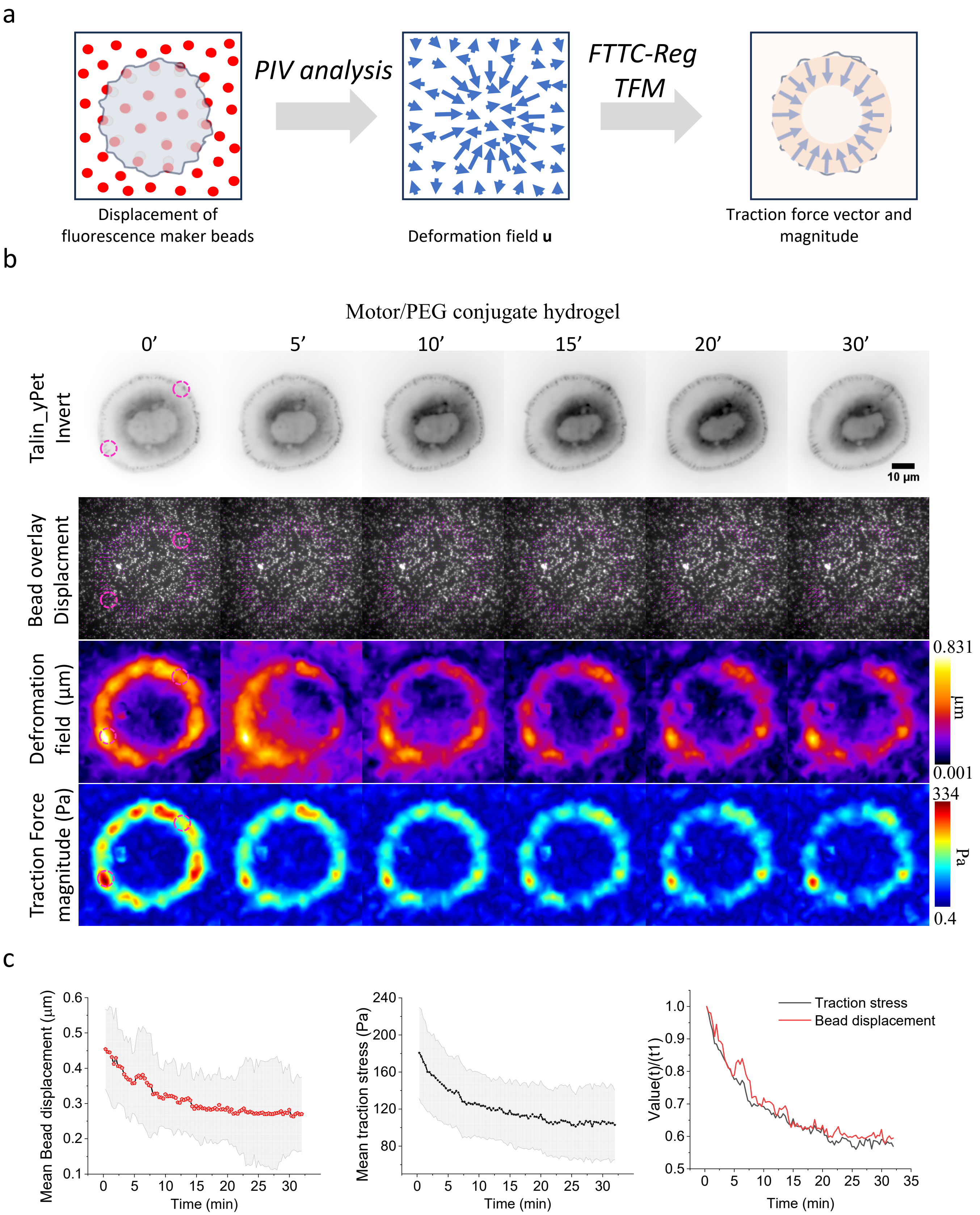
a
PIV analysis
FTTC-Reg
TFM
Displacement of fluorescence maker beads
Deformation field **u**
Traction force vector and magnitude
b
Motor/PEG conjugate hydrogel
0'
5'
10'
15'
20'
30'
Talin_yPet Invert
10 μm
Bead overlay Displacment
Defromation field (μm)
0.831
μm
0.001
Traction Force magnitude (Pa)
334
Pa
0.4
c
Mean Bead displacement (μm)
Time (min)
Mean traction stress (Pa)
Time (min)
Value(t)/(t1)
Traction stress
Bead displacement
Time (min)

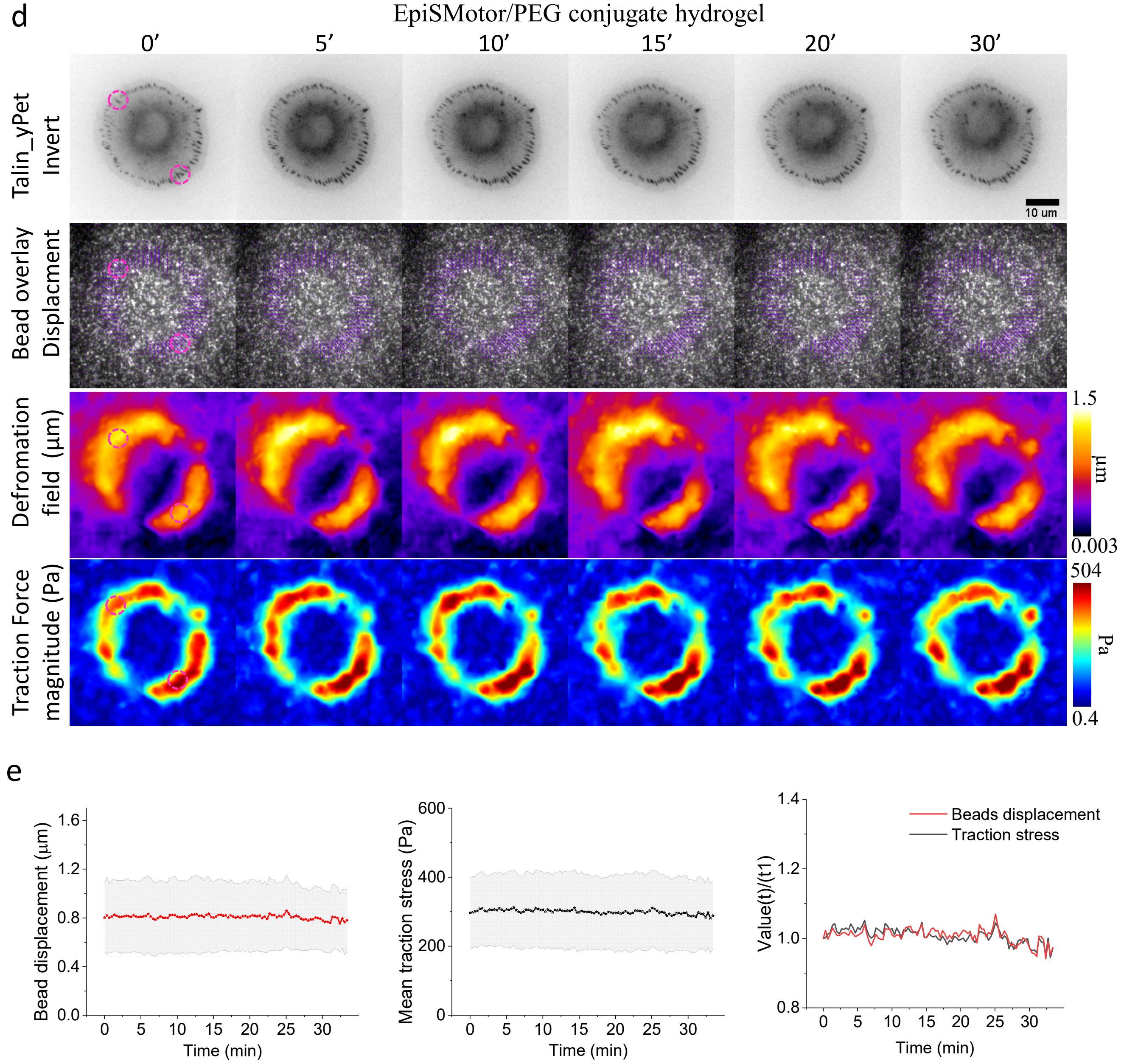

Figure s12: Validate of the accuracy of the traction force reconstitution from bead displacements a) Diagram illustrating the method for measuring traction force. **b)** MEK fibroblast cells expressing talin-YPet, cultured on a motor/PEG conjugate containing platform with an actuated hydrogel, illuminated by a UV laser and marked with a dotted region of interest at various time intervals indicated above. The corresponding deformation field and traction force measure throughout the actuation process. **c)** The total displacement of the cell divided by the cell area is plotted during the actuation of two ROIs, with traction force also plotted. Finally, the data is normalized to t=0 to facilitate a comparison of the rates of reduction in both displacement and traction force during the actuation process, ensuring that there is no alteration in the relationship between displacement and traction force reconstitution. **d) - e)** Data similar to that presented in panels b, c, and d, except that the cells were cultured on EpiSMotor containing hydrogel, which serves as a non-rotatory control condition.

Figure S13

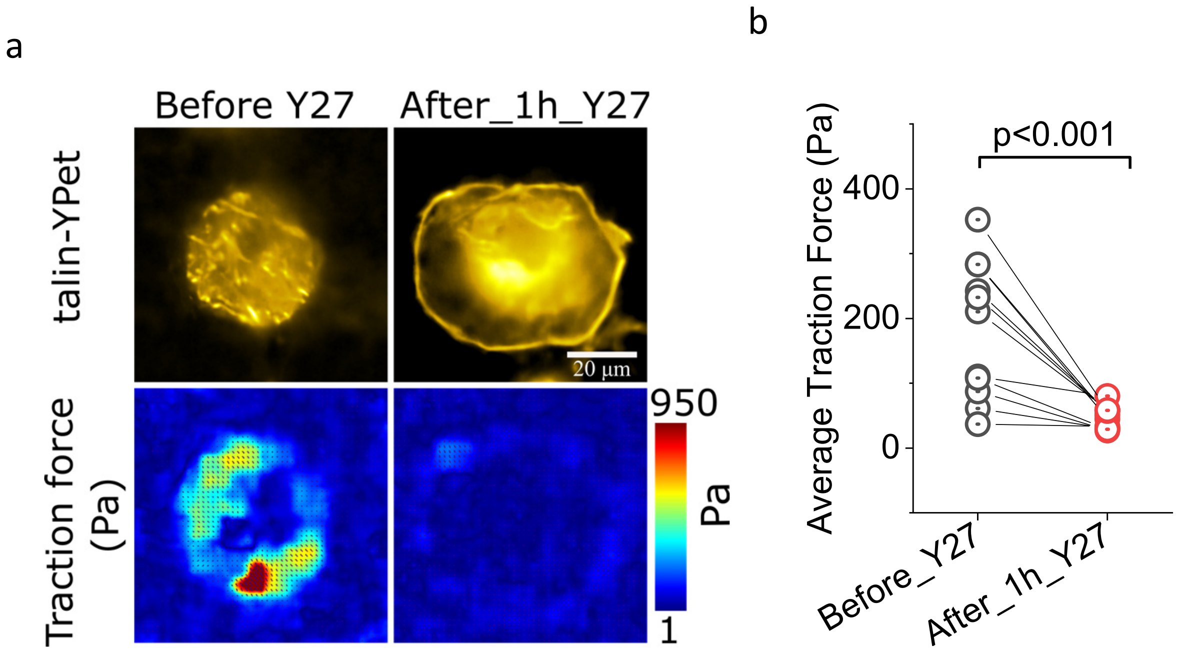

Figure s13: Effect of 5 µM Y27632 drugs treatment for 1h on cell traction force .
Talin-YPet re-expressing MEK fibroblast on motor/PEG/RGD/Bead was treated with 5 µM Y27632 drugs for 1h. **a)** Talin-YPet and corresponding traction force image of same cells before and 1h after drugs treatment. **b)** Line series plot of average traction force of cell tracked before and after 5 µM Y27632 treatment. Data represent $n = 11$ cells. Statistical significance was assessed using the Wilcoxon matched-pairs signed rank test ($p < 0.001$).

Figure S14

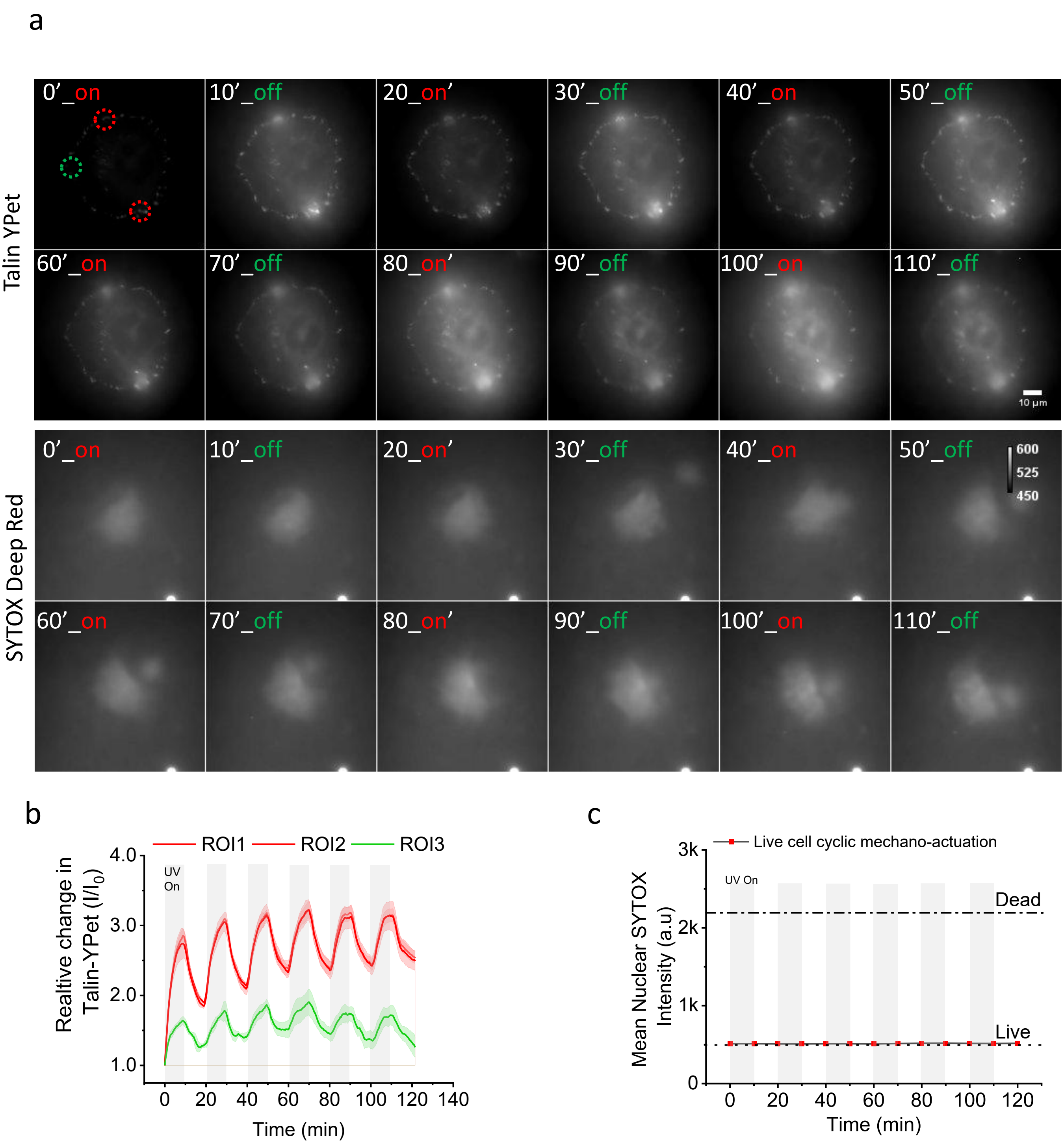

Figure s14: Long term cyclic mechano-actuation and evaluation of cellular vitality (membrane integrity) in MEK fibroblasts . a) Representative images of Talin-YPet MEK fibroblast cells grown on motor/PEG//RGD hydrogels interface subjected to cyclic illumination with on/off intervals of 10 min and the corresponding SYTOX intensity image. **b)** Line scatter plot showing the average Talin-YPet intensity over 120 minutes of cyclic mechano-actuation (mean ± s.d. from two independent experiments). **c)** Plot of SYTOX intensity over the course of cyclic mechano-actuation (mean ± s.d. from two independent experiments).
